# A Comprehensive Review of Edible Flowers with a Focus on Microbiological, Nutritional, and Potential Health Aspects

**DOI:** 10.3390/foods14101719

**Published:** 2025-05-12

**Authors:** Angela Daniela Carboni, Tiziana Di Renzo, Stefania Nazzaro, Pasquale Marena, Maria Cecilia Puppo, Anna Reale

**Affiliations:** 1 Center for Research and Development in Food Science and Technology, Consejo Nacional de Investigaciones Científicas y Técnicas, La Plata RA1900, Argentina; mcpuppo@quimica.unlp.edu.ar; 2 Institute of Food Sciences, National Research Council, ISA–CNR, 83100 Avellino, Italy; tiziana.direnzo@isa.cnr.it (T.D.R.); stefania.nazzaro@isa.cnr.it (S.N.); pasquale.marena@isa.cnr.it (P.M.)

**Keywords:** bacteria, pathogens, antioxidants, polyphenols, health benefits, novel ingredients, natural colorants, toxicity

## Abstract

Edible flowers have been used since ancient times directly as food, flavoring agents, and garnish in food products, and are now reappearing in modern cuisine. Edible flowers have gained popularity due to changing consumer habits focused on healthier food options. In addition to contributing to the esthetics and flavor of various dishes, edible flowers are now recognized for their nutritional value, as they contain bioactive components with different health benefits. However, a significant concern regarding edible flowers is the potential contamination by undesirable microorganisms. Since edible flowers are often consumed fresh or minimally processed, they can pose a microbiological risk. Edible flowers may be susceptible to contamination by various pathogenic microorganisms, particularly *Bacillus* spp., *Enterobacter* spp., *Salmonella* spp., and *Staphylococcus aureus.* In addition, mycotoxin-producing fungi, such as *Aspergillus*, *Penicillium*, *Alternaria*, or *Fusarium*, can be found in various flowers. Good agricultural practices, hygienic handling, and appropriate storage are essential to reduce contamination and guarantee the safe consumption of edible flowers. Since current investigations on the microbiological safety aspects of edible flowers are scarce, this review aims to provide an overview of the consumption of edible flowers and a discussion of their uses, health benefits, and risks, focusing on microbiological aspects.

## 1. Introduction

Current food trends include the search for products perceived as ‘natural’ and with certain health-promoting properties [1,2,3]. In addition, plant-based foods have also become more popular in recent years [1]. Part of these trends can include edible flowers, considered as those flowers that are harmless and safe to eat and that bring positive health effects to the human being [4,5]. Edible flowers have been used since ancient times by different cultures for various purposes, including nutritional value, medicinal properties, sensory qualities, and ornamental value [6,7]. Although these are foods with a long history of use, recent years have seen a resurgence in the consumption of edible flowers [6,8,9]. In some countries, it is possible to purchase edible flowers in various kinds of shops, including supermarkets and health food stores, while in other nations the flower market is still limited to the production of ornamental ones [8,10].

The use of edible flowers in food production allows us to obtain products with distinctive color, appearance, and flavor features, making them interesting ingredients for the preparation of gourmet dishes, where visual perception and esthetics play an important role [1,2,11,12]. In addition, several research topics focus on finding natural alternatives to the use of food additives [13]. In this context, natural pigments present in edible flowers can be considered useful to providing color to beverages, yogurts, baked goods, and desserts [13,14,15,16]. As an example, innovative results can be obtained by adding certain flowers to food matrices, such as *Clitoria ternatea* (known as pea flower). This flower can add blue tones to food products and exhibits remarkable color-changing properties depending on the pH of the medium, shifting shades in acidic or alkaline environments [17]. Despite the importance of food appearance in influencing consumer’s purchasing decisions, health consciousness plays a role in the consumption of edible flowers, so it is interesting to evaluate the amounts and forms of preparation required for each edible flower to maximize health benefits [12,18].

Edible flowers are recognized for their potential health-promoting properties including high antioxidant capacity, anti-bacterial effects, and a nutritional composition characterized by a wide variety of vitamins and minerals and a low-calorie content [19,20]. However, some of these flowers contain natural compounds that, while beneficial for the plant’s defense, may have adverse effects on the human health. Furthermore, these foods can be associated with different biological (e.g., microorganisms, insects, etc.) or chemical risks [2,21]. On the other hand, the diversity of edible flowers can make it challenging to distinguish between toxic and non-toxic species. Regarding the microbiological characteristics of edible flowers, they can be contaminated by different microorganisms, including species belonging to the genera *Salmonella*, *Bacillus*, *Pseudomonas*, and *Enterobacter*, and this contamination can occur before or during harvest or also during the post-harvest handling of the product [18]. To date, scientific research on the microbiological safety aspects of edible flowers is scarce, so further studies are needed to assess the factors that may influence their safety in order to promote their marketing and consumption and extend shelf-life [8].

This review aims to bring together the current knowledge on the different aspects of edible flowers, with a particular focus on their microbiological aspects, nutritional attributes, and health benefits as well as their safety characteristics. The information provided is intended to promote the intake of safe edible flowers while also exploring the most effective methods of preparation.

## 2. Varieties of Edible Flowers

**Figure 1:**
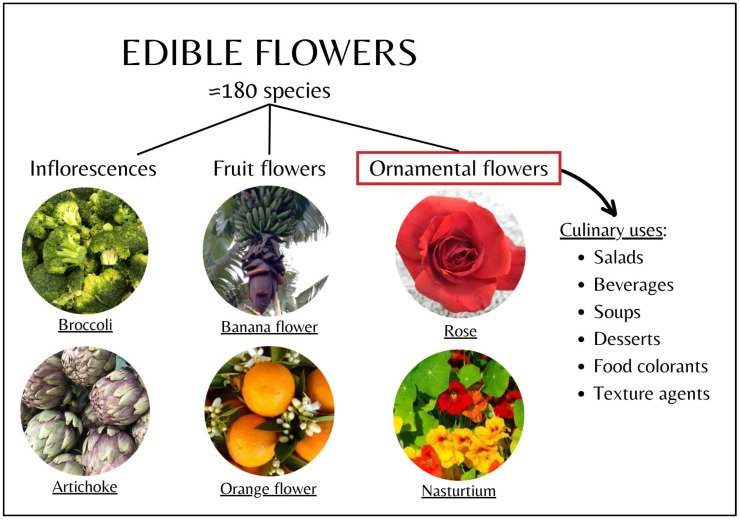
Classification and culinary uses of edible flowers. This review focuses on the use of ornamental flowers as edible flowers.

The flowers that are considered edible include approximately 180 species [22]. This large number comprises a wide variety of flowers, which can be categorized in different ways. One classification of edible flowers can be into fruit flowers (e.g., citrus or banana flowers) and non-fruit (or ornamental) flowers (e.g., hibiscus, begonias, and calendula) [8,23]. Additionally, some foods considered vegetables are actually inflorescences (e.g., artichoke, broccoli, and cauliflower) [3]. Figure 1 shows the different types of edible flowers. In this review, only ornamental flowers will be discussed.

In particular, the best-known species of ornamental edible flowers, their scientific and common names, their color and sensory characteristics, and their applications as food additives or for traditional and culinary uses are summarized in Table 1. As can be seen, most edible flowers are currently used in the preparation of different types of beverages and infusions or are included in salads or soups [8].

Chive flowers (*Allium schoenoprasum* L.), for example, are often used in dishes preparation since they offer a mild onion-garlic flavor with a hint of floral notes [3,24]. Species such as *Begonia × tuberhybrida* and *Begonia × semperflorens-cultorum*, *Hibiscus* L., and *Pelargornium hortorum*, are known for their citrus-like sensory taste and vibrant color and are often added to salads or used as garnish for dishes and drinks [3,7,25,26,27,28,29,30,31]. Flowers of *Chrysanthemum* spp., *Cichorium intybus* L., *Tagetes erecta*, *Taraxacum officinale* Weber, *Tropaeolum majus* L., and *Viola tricolor* are characterized by a bitter flavor and are widely used in both sweet preparations (pancakes, cookies, and desserts) and savory dishes (salads and omelets), making them a valuable ingredient in various cuisines, mainly Asian [25,26,30,32,33,34,35,36,37]. Then, there are flowers that have a sweeter, honey-like or intense floral flavor such as *Bougainvillea glabra*, *Lavandula angustifolia, Jasminum sambac, Malva sylvestris*, *Matricaraia chamomilla*, *Tulip gesneriana* L., or *Viola × wittrockiana*, that are used in the preparation of beverages, dessert, infusions, or syrups [26,27,28,30,38,39,40,41].

In general, edible flowers possess a delicate and non-invasive flavor. However, some have bitter or spicy notes that may not be appreciated by consumers. Some edible flowers may even resemble other foods, which facilitates their acceptance. Benvenuti et al. [42] conducted a sensory panel on this matter. Tasters were asked to associate the flavor of various flowers with foods and the panelists found similarities between certain flowers and fruits (*Begonia* was found like lemon, while *Tagetes erecta* resembled pomegranate) and vegetables (*Borago officinalis* L. was found like cucumber and *Tropaeolum majus* L. to radish). In addition, Cheng et al. [43] found that volatile compounds can differ according to the organ of the flower, with the petals having the highest content of these compounds in some cases.

Despite some studies having addressed this topic, further scientific research on sensory aspects of different flower varieties is needed to better understand their possible acceptance by consumers. An online questionnaire regarding the intake of edible flowers, carried out by Mulik et al. [44], revealed that almost 90% of the participants had a positive experience in consuming these kinds of flowers. This provides further exploration in this field, suggesting that edible flowers have the potential to achieve high levels of approval from the general population.

Furthermore, special attention can be given to the use of these flowers as additives in the food industry, since some of them can act as natural coloring agents (e.g., *Bougainvillea glabra*, *Clitoria ternatea*, and *Hibiscus* L.) or as texture agents (e.g., *Hibiscus* L. and *Malva sylvestris* L.) [45,46,47]. *Bougainvillea glabra* is known for its vibrant pigments and it is often used as a natural food coloring agent. *Clitoria ternatea*, instead, is rich in anthocyanins, and provides a striking blue or purple color to beverages, desserts, and other foods [47]. Also, *Hibiscus* L. contains anthocyanins and serves as a natural red or pink food dye, commonly used in teas, syrups, jams, and desserts [30]. Flowers like *Hibiscus* L. and *Malva sylvestris* are rich in mucilage which acts as natural thickener in soups, sauces, and desserts [48,49].

**Table 1:** Sensory aspects and food application of different edible flowers.

Scientific Name	Common Names	Color	Sensory Features	Food Application	Countries of Consume	References
*Allium schoenoprasum* L.	Chives	Pink, purple	Onion taste, strong flavor, spicy	Dips, soup	Europe, Asia, India	[3,24]
*Begonia × tuberhybrida*	Tuberous begonia	Pink, red, white, yellow	Citric and sweet flavor, crisp texture	Salads	Unknown	[25,26,28]
*Begonia × semperflorens-cultorum*	Wax begonia	Red, white	Citric, sour and sweet flavor, crisp texture	Salads	Unknown	[7,25,26,27]
*Borago officinalis* L.	Borage, starflower	Blue, purple, white	Similar flavor to cucumber, sweet flavor	Drinks, salads, soups, used as a spice	Denmark, Italy, Spain	[3,42,50,51]
*Bougainvillea glabra*	Bougainvillea, Bougainville, glory of the garden, paper flower	Orange, pink, violet	Intense color, subtle flavor	Food colorant, infusions, lemonade, salads	Thailand	[30,34,38,45,52]
*Calendula officinalis* L.	Calendula, pot marigold	Orange, yellow	Slightly bitter, similar flavor to saffron, peppery, easy chewiness	Beverages, food colorant infusions, salads	Bosnia-Herzegovina, Brazil, France, Italy	[3,25,37,42,50]
*Centaurea cyanus* L.	Centaurea, cornflower, bachelor’s buttons	Blue	Spicy, vegetal flavor	Cakes, cookies, desserts, food colorant, infusions, salads	Unknown	[26,27,30]
*Chrysanthemum* spp.	Chrysanthemum	Pink, purple, white, yellow	Bitter flavor	Desserts, infusions, salads, soups	China	[26,32,53]
*Cichorium intybus* L.	Chicory	Blue, violet	Bitter flavor, similar flavor to endive	Salads, soups	Unknown	[3,27]
*Clitoria ternatea*	Asian pigeon wings, blue bell vine, blue pea flower, butterfly pea, Darwin pea	Blue, violet	Color changes with pH	Bio-preservative, bread, cakes, chocolate, food colorant, fermented beverages, infusions, noodles, yogurt	Argentina	[14,15,17,46,47,54,55,56,57,58]
*Cucurbita pepo* L.	Zucchini flower	Orange, yellow	Slightly sweet, mild aroma	Dressings, fried, salads, soup, stuffed	Italy, Mexico, Slovenia, Spain, Türkiye	[9,50,59,60,61,62]
*Dahlia mignon*	Dahlia	Pink, white, yellow	N.D.	Food colorant	Mexico	[13,63]
*Hibiscus* L.	Hibiscus, Jamaica tea flower	Pink, red, orange	Acid and citrus flavor	Beverages, cakes, desserts, food colorant, ice cream, jam, muesli, pickles, salads, sauces, syrup, texture agent	China, Taiwan	[3,29,30]
*Jasminum sambac*	Arabian jasmine, Asian jasmine	White	Intense and floral aroma	Beverages, desserts, flavor agent, infusions, syrup	Taiwan	[12,30,41,64,65]
*Lavandula angustifolia*	Lavender	Lavender, purple, violet	Highly perfumed, intense flavor	Beverages, cakes, desserts, dressings, ice-cream, jam, pastry, soups	Argentina, Italy, Spain, Taiwan	[12,30,50]
*Malva sylvestris* L.	Blue Mallow, blue malva, cheese flower, common mallow	Pink, violet, white	Honey-like, sweet and floral flavor, spicy	Edible coating, food colorant, infusions, salads, soups, thickener	Boznia-Herzegovina, Spain	[30,39,50,66]
*Matricaria chamomilla*	Chamomile, German chamomile	White, yellow	Floral flavor, sweet scented	Beverages, desserts, baked goods, ice cream, infusions, jam, salads	Unknown	[27,40,67]
*Nelumbo nucifera*	Lotus	White	Crunchy texture, mild flavor	Infusions, fried, salads, soups	Vietnam	[27,30,34,40]
*Pelargonium hortorum*	Common geranium, garden geranium, malvon	Orange, pink, red, white	Citric flavor	Beverages, desserts, salads,	Unknown	[31]
*Petunia hybrida*	Petunia	Blue, pink, purple, red, white, yellow	Mild- tasting	Salads	Unknown	[30]
*Rhododendron arboreum*	Rhododendron	Pink, red, white	Sweet, sour flavor, bitter	Fermented beverages, food colorant, flavor agent, jam, jellies, juices, yogurt	China	[30,68,69,70,71,72]
*Rosa* spp.	Rose	Orange, red, pink, white, yellow	Astringent, highly aromatic, slightly bitter, sweet	Beverages, desserts, food colorant, ice cream, infusions, jam, liquors, muesli, preservative in meat products, syrup	Brazil, China, Taiwan, Slovenia	[2,3,12,25,26,28,29,60,73]
*Tagetes erecta*	Marigol, Mexican marigold	Orange, red, white, yellow	Bitter, similar flavor to pomegranate, strong flavor	Baked goods, beverages, butter, salads, soups	Mexico, Thailand	[3,27,33,34,42]
*Taraxacum officinale* Weber	Dandelion, Lion’s Tooth	Yellow	Bitter flavor	Desserts, cheese, flavor agent in sweet meals, salads, vegan honey substitute, wine	Unknown	[3,35,36]
*Tropaeolum majus* L.	Empress of India, nasturtium, monks Cress,	Orange, red, white, yellow	Bitter, peppery, similar flavor to radish, spicy	Beverages, dips, salads, vinegar	Denmark, Croatia	[25,30,37,42,50,51]
*Tulip gesneriana* L.	Tulip	Red, pink, yellow,	Similar flavor to pea, sweet,	Salads, stuffed, syrup	Unknown	[28,30]
*Viola tricolor* L.	Heart’s ease, wild pansy	Multi-colored, pink, violet, white, yellow	Bitter	Beverages, cookies, desserts, salad, soups	Australia, Denmark, Italy	[3,27,30,50,51]
*Viola × wittrockiana*	Pansy	Multi-colored, pink, violet, yellow, white	Sweet flavor	Beverages, cookies, desserts, salad, soups	Brazil	[3,26,30,60]

## 3. Nutritional Composition of Edible Flowers

Edible flowers are increasingly recognized for their nutritional and health-promoting properties, being rich in essential nutrients, bioactive compounds, and phytochemicals that contribute to a balanced diet and provide various functional benefits. The chemical composition and bioactive constituents of edible flowers are shown in Table 2. According to Jadhav et al. [8], water is the major component of edible flowers (about 70–90% of the total weight); so, the dry matter in edible flowers is very low. Therefore, due to their low yield, a significant amount of fresh flowers is required to produce 100 g of dried flowers [34,74].

The proximate composition of edible flowers varies greatly between species, but several general trends can be observed. Edible flowers are a good source of protein and dietary fiber, with protein content typically ranging from 2% to 23%, depending on the species and environmental factors affecting growth [25,75]. The lipid content is generally lower than 10%, while soluble sugars, composed by total or reducing sugars, are present in amounts higher than 15% [10,25,75,76,77]. These sugars contribute to the sweetness and flavor profile of certain flowers. Crude fiber (CF) content varies from 0.4% to 20.5%, while total dietary fiber (TDF) values range from 17.2% to 75.9% [10,75,78,79]. TDF values are usually more than 50% higher than CF values, because they include non-digestible molecules such as polysaccharides and oligosaccharides (e.g., cellulose, lignin, pectin, mucilage, and gums) that provide low amounts of calories. Finally, the ash content, which represents the total minerals of edible flowers, has been reported to range from 3.8% to 22.0% with some exceptions depending on the specific type of flower [76,78]. Other nutritional components of edible flowers include a variety of phenolic compounds, vitamins, and minerals, as well as flavonoids and carotenoids. Specific characteristics of the major compounds present in edible flowers are detailed below.

### 3.1. Carbohydrate, Sugar, and Fiber Composition

Carbohydrates are one of the most abundant compounds in edible flowers, with reported values exceeding 90 g/100 g of dry weight (d.w) in species such as *Rosa spp.* [80] or 80 g/100 g in *Hibiscus acetosella* [81]. The total carbohydrate content can vary from 3.3 to 90.0% depending on the flower species.

The composition of soluble sugars in edible flowers is crucial for their taste and nutritional value. Fructose, glucose, and sucrose are the primary sugars present in flowers, with significant variability depending on the species [82]. As reported in Table 2, different authors found high amounts of total sugars in flowers from *Allium* spp., *Borago officinalis*, *Centaurea cyanus*, *Cucurbita moschata (*Butternut squash), and *Fuchsia regia*.

Regarding dietary fiber content of edible flowers, Jakubczyk et al. [83] evaluated different flower varieties. Among them, samples of *Calendula officinalis* L. showed the highest total fiber content (62.3 g/100 g d.w), followed by *Centaurea cyanus* L. (53.1 g/100 g d.w), *Cichorium intybus* L. (32.2 g/100 g d.w), *Taraxacum officinale* (27.0 g/100 g d.w), *Syringa vulgaris* L. (25.9 g/100 g d.w), and *Magnolia × soulangeana* (13.2 g/100 g d.w). Most of the fiber present in these samples corresponded to insoluble fiber, with values ranging from 8.7 g/100 g d.w (*Magnolia × soulangeana*) to 57.5 g/100 g d.w (*Calendula officinalis* L.).

**Table 2:** Nutritional composition of different edible flowers (d.w).

Scientific Name	CarboH (g/100 g)	Sugars (g/100 g)	Proteins (g/100 g)	Lipids (g/100 g)	Fiber (g/100 g)	Ash (g/100 g)	Minerals ^1^ (mg/100 g)	Vitamin C (mg/100 g)	TPC—CAR	References
*Allium* spp.	3.3–18.6	6.6–26.2	-	-	-	-	-	35.4–157.8 (f.w)	TPC: 3.6–10.6 CAR: 3.0–23.4	[84]
	-	50.0	15.3	3.4	CF: 6.1	3.8	-	542.1	TPC: 1877.9 CAR: 291.1	[76]
	-	-	-	-	-	-	Potassium (K) Calcium (Ca), Sodium (Na), Iron (Fe), Zinc (Zn)	-	-	[85]
*Begonia × tuberhybrida*	-	-	3.9	-	-	-	K, Ca, Na	-	TPC: 42.3	[25]
*Borago officinalis* L.	- -	28.8	9.4	4.3	TDF: 40.4	9.3	-	-	-	[10]
	-	16.8	22.7	4.9	TDF: 35.4	15.3	-	-	CAR: 181.4	[75]
	-	20.3	14.4	-	CF: 15.3	14.7	-	196.4	-	[77]
	-	-	-	-	-	-	K, Na, Ca, Fe, Zn, Manganese (Mn)	-	TPC: 16.6 TF: 12.6	[84]
*Calendula officinalis* L.	3.6 (f.w)	6.2 (f.w)	4.6 (f.w)	-	CF: 1.1 (f.w)	18.4	Fe, Zn, Mn	40.0 (f.w)	TPC ^2^: 61.0 (f.w) TF: 37.9 (f.w)	[79]
	-	-	-	-	-	-	K, Na, Ca, Fe, Zn, Mn	-	TPC: 16.3 TF: 9.4	[86]
	-	-	3.9	-	-	-	K, Ca, Na	-	TPC: 40.6	[25]
	-	-	8.7	-	TDF: 62.3	-	-	-	-	[83]
	-	-	-	-	-	-	-	-	TPC: 290.8 CAR: 5745.3	[87]
*Centaurea cyanus*	-	11.9	8.5	4.4	TDF: 75.9	5.7	-	-	-	[10]
	-	20.6	6.9	3.4	TDF: 67.4	5.2	-	-	CAR: 5.8	[75]
	-	-	6.9	-	-	-	K, Ca, Na	-	TPC: 48.9 TF ^3^: 18.6	[26]
	-	-	9.6	-	TDF: 53.1	-	-	-	-	[83]
*Chrysanthemum frutescens*	-	-	7.2	-	-	-	K, Ca, Na	-	TPC: 26.4 TF ^3^: 12.9	[26]
*Cucurbita pepo*	-	-	21.9	5.0	10.5	15.9	-	-	-	[88]
*Cucurbita máxima* spp.	-	-	-	24.8	41.4	28.1	-	0.4	TPC: 498.3 TF: 304.4	[89]
	25.1	-	14.8	17.0	CF 20.5	22.0	-	149.2	-	[78]
	-	-	-	-	-	-	Ca, K, Na, Fe, Zn	-	-	[85]
	5.3 (f.w)	2.0 (f.w)	2.2 (f.w)	0.2 (f.w)	CF 4.4 (f.w)	3.1 (f.w)	K, Ca, Na, Fe	-	-	[90]
*Cucurbita moschata* Duchesne	-	-	14.5	-	-	-	Ca, Mn, Fe, Zn	10.7	TPC: 8.4 TF: 3.8 CAR: 8.8	[91]
*Dianthus chinensis* L.	-	-	9.7	-	-	-	Ca, Fe, Mn, Zn	122.1	TPC: 10.1	[91]
	32.6 (f.w)	12.1 (f.w)	19.5 (f.w)	-	CF: 1.4 (f.w)	6.1	Fe, Mn, Zn	100.0 (f.w)	TPC: 52.5 (f.w)	[79]
	-	-	-	-	-	-	K, Ca, Na, Fe, Zn	-	-	[92]
	-	-	-		-	-	-	-	TPC: 179.6–248.6 CAR: 49.1–75.9	[87]
*Fuchsia regia*	-	-	6.1	-	-	-	Ca, Fe, Zn, Mn	44.0	TPC: 148.8	[91]
*Fuchsia × hybrida*	-	-	2.8	-	-	-	K, Ca, Na	-	TPC: 41.2 TF ^3^: 19.8	[26]
*Hibiscus acetosella*	83.6	-	-	10.9	-	5.5	-	-	-	[81]
*Lavandula angustifolia*	-	-	-	-	-	-	-	-	TPC:14.8–32.8 TF:8.5–23.7	[93]
	-	-	-	-	--	-	-	9.0	TPC: 12.7	[37]
		10.9	11.5	-	CF: 17.6	7.3	-	110.3	-	[77]
	-	-	-	-	-	-	K, Ca, Na, Fe, Mn, Zn	-	TPC: 17.3 TF: 18.6	[86]
*Nyctanthus arbortristis*	-	-	-	-	49.7	-	-	0.7	TPC: 1486.2 TF: 660.2	[89]
*Pelargonium hortorum*	41.8 (f.w)	5.2 (f.w)	16.3 (f.w)	-	CF: 0.9 (f.w)	7.4	Fe, Zn, Mn	42 (f.w)	TPC: 108.0 (f.w)	[79]
*Petunia hybrida*	18.4 (f.w)	2.4 (f.w)	15.3 (f.w)	-	CF: 2.10 (f.w)	14.7	Fe, Zn, Mn	28.0 (f.w)	TPC: 50.5 (f.w)	[79]
*Rosa odorata*	-	-	2.6	-	-	-	K, Ca, Na	- - -	TPC: 49.8 TF ^3^: 20.2	[26]
*Rosa micrantha*	90.2	13.1	4.3	1.3	-	4.2	-	295.1	TPC: 424.2 TF: 78.5 CAR: 46.6	[80]
*Rosa* spp.	-	-	2.0	-	-	-	K, Ca, Na	-	TPC: 30.9	[25]
	-	-	-	-	-	-	K, Ca, Na, Fe, Zn, Mn	-	TPC: 9.9 TF: 2.6	[86]
*Syringa vulgaris* L.	-	-	12.4	-	TDF: 25.9	-	-	-	-	[83]
*Tagetes patula/erecta*	-	-	3.2	-	-	-	K, Ca, Na	-	TPC: 51.2	[25]
	-	-	3.0	-	-	-	K, Ca, Na	-	TPC: 47.5 TF ^3^: 19.6	[26]
	-	-	-	-	-	-	-	-	TPC: 194.8–303.6 CAR: 500.6–2057.8	[87]
*Taraxacum officinale*	-	-	13.2	-	TDF: 27.0	-	-	-	-	[83]
*Tropaeolum majus*	-	-	6.2	-	-	-	K, Ca, Na	-	TPC: 43.8	[25]
	-	-	4.2	-	-	-	K, Ca, Na	-	TPC: 29.3 TF ^3^: 45.4	[26]
	-	-	-	-	-	-	K, Ca, Na, Fe, Zn, Mn	-	TPC: 23.0 TF: 5.1	[86]
*Viola cornuta* L.	-	-	12.9	-	-	-	Ca, Zn, Mn, Fe	248.8	TPC: 33.9	[91]
*Viola tricolor* L.	-	10.28	13.3	-	CF: 8.4	16.7	-	577.7	-	[77]
	-	-	-	-	-	-	K, Ca, Na, Fe, Zn, Mn	-	TPC: 63.4 TF: 32.8	[86]
*Viola wittrockiana*	11.8 (f.w)	8.0 (f.w)	2.3 (f.w)	-	CF: 0.4 (f.w)	3.2	Fe, Zn, Mn	32 (f.w)	TPC: 13.9 (f.w)	[79]
	-	27.9-W 8.53-Y 10.4-R	23.3-W 15.3-Y 9.1-R	5.2-W 9.7-Y 4.5-R	TDF: 17.2-w TDF: 32.0-Y TDF: 25.4-R	10.6-W 8.2-Y 6.3-R	-	-	CAR: 21.6-W 58.0-Y 109.2-R	[75]

CarboH: carbohydrates; TDF: total dietary fiber; CF: crude fiber; f.w: fresh weight; Viola W: white; Y: yellow; R: red; TPC: total polyphenol content (mg GAE/g); CAR: carotenoids (mg/100 g); TF: total flavonoids (mg/g). ^1^ Listed according to amounts, from major to minor. TPC ^2^: total polyphenol content expressed as mg catechol/g fw. TF ^3^: Total flavonoids expressed as mg rutin/g.

On the other hand, the amounts of soluble fiber are noticeably lower than those of insoluble fiber, with the lowest value of 1.4 g/100 g d.w in *Syringa vulgaris* and the highest of 7.5 g/100 g d.w in *Centaurea cyanus* L. 

### 3.2. Protein Composition

Among the various edible flowers, *Borago officinalis* L. is one of the flowers with the highest reported protein content (approximately 22.7 g/100 g d.w) [75] (Table 2). Other notable protein sources are *Cucurbita pepo* (Zucchini) and *Cucurbita moschata* (Butternut squash), with values ranging from 14.5 to 21.9 g/100 g (d.w) [88,91]. Also, flowers like *Dianthus chinensis, Pelargonium hortorum*, *Petunia hybrida, Syringa vulgaris* L., *Viola cornuta*, and *Viola wittrockiana* (white variety) have more than 13% protein (d.w) [75,83,91]. In contrast, petals from Rosa species generally have low amounts of proteins, often below 5%. Also, flowers of *Fuchsia regia* (6.1% d.w) and *Fuchsia × hybrida* flowers (2.8% d.w) are characterized by low protein levels [26,91].

### 3.3. Mineral and Vitamin Composition

Edible flowers are also a notable source of essential minerals. The main macroelements identified in flowers are calcium (Ca), sodium (Na), and potassium (K) while among microelements, iron (Fe), manganese (Mn), and zinc (Zn) have been identified (Table 2). Other commonly determined minerals include phosphorous (P), magnesium (Mg), and copper (Cu). Flowers generally contain high amounts of Ca, although concentrations vary between species. For instance, values range from 74 mg/100 g in *Cucurbita pepo* to 9050 mg/100 g in *Fuchsia regia* [88,91]. Regarding microelements, the amount of Fe is remarkably higher than Zn in all the flowers listed in Table 2, except for *Viola cornuta.* Notably*, Dianthus chinensis* and *Petunia hybrida* have high iron contents with 133 mg/100 g and 173 mg/100 g of fresh weight (f.w), respectively [79]. Edible flowers also presented remarkable contents of K, especially in *Calendula officinalis* L., *Lavandula angustifolia*, and *Tropaeolum majus,* with values higher than 4000 mg/100 g (d.w) [86].

### 3.4. Phenolic Compounds and Other Bioactive Substances

As described before, edible flowers are increasingly recognized for their rich composition in phenolic compounds and other bioactive substances, contributing to their moderate to high antioxidant activity [20]. The information about the total polyphenol content, total flavonoids, and carotenoids in edible flowers is included in Table 2.

Phenolic compounds, as highlighted by Zheng et al. [94], can have numerous health benefits, being characterized by antioxidant, anti-inflammatory, neuroprotective, hepatoprotective, and anti-diabetic properties. Studies have suggested that new compounds such as polysaccharides, lignans, and phenolic glycosides also contribute to these health benefits. Edible flowers, particularly those from the *Allium* family, are increasingly recognized for their nutritional and health benefits [95]. The most commonly cultivated edible flowers of *Allium* species include *Allium schoenoprasum* L. (chive), *Allium sativum* L. (garlic), *Allium cepa L*. (onion), and *Allium ampeloprasum* L. (leek), among others. These plants are recognized for their anti-bacterial, antioxidant, anti-inflammatory, and anti-proliferative properties [96,97]. In a study by Grzeszczuk et al. [76], *Allium* spp. presented a notably high total phenolic content (1877 mg GAE/g d.w). Chetia et al. [89] also demonstrate that *Nyctanthus arbortristis* (Night jasmine) and *Cucurbita máxima* (pumpkin) possess an interesting phenolic content (1486 and 498 mg GAE/g d.w, respectively). High antioxidant activity was also recorded in different flowers such as *Tagetes erecta* (70.4 mol FeSO_4_/100 g f.w), *Fuchsia hybrida* (47.5), *Dianthus barbatus* (38.6), *Viola × wittrockiana* (36.5), and *Pelargonium peltatum* (34.7), as reported by Benvenutti et al. [7]. In contrast, lower values of antioxidant activity (below 10 µmol FeSO_4_/100 g f.w) were observed in *Borago officinalis*, *Calendula officinalis*, white *Dianthus barbatus*, and various cultivars of *Petunia × hybrid* and *Viola × wittrockiana.*

As reported by dos Santos et al. [81], *Hibiscus acetosella*, a flower widely consumed in Brazil, is rich in several compounds with bioactive functions, including high antioxidant activity. Part of these components comprise substances like different anthocyanins, gallic acid, caffeic acid, and quercetin, among others. Also, other non-common edible flowers such as *Bellis perennis*, *Rumex acetosa*, *Salvia pratensis*, *Sambucus nigra*, *Tragopogon pratensis*, *Trifolium repens*, and *Viola arvensis* are characterized by their high phenolic compound composition and promising antioxidant activity [98].

Despite the high levels of antioxidants present in the different edible flowers, these compounds can be sensitive to environmental conditions, such as light, heat, and oxygen, particularly during post-harvest handling. Therefore, understanding how different storage methods and environmental factors impact their nutrient composition is crucial for maintaining their health benefits and extending their shelf-life.

## 4. Health Benefits of Edible Flowers

The possible health benefits and risks associated with the consumption of different edible flowers are summarized in Table 3. The evidence supporting these effects is derived from in vitro studies and clinical observations. This distinction should be kept in mind when interpreting the potential health benefits and risks associated with edible flower consumption. The bibliographic reference to each potential benefit was added.

The main health benefits of incorporating edible flowers into diets are related to their antioxidant, anti-proliferative, anti-diabetic, anti-obesity, and cardio-protective properties [99]. The antioxidant activities of edible flowers can be explained by the presence of different amounts of phenolic compounds, and among them, their flavonoid content (See Section 3.4) [18]. By protecting the human body from oxidative stress, these compounds can help to improve immune function, enhance anti-inflammatory effects, and reduce the risk of chronic pathologies [23,100,101].

Other common benefits of flower ingestion include anti-carcinogenic, anti-diabetic, and anti-bacterial properties. Antioxidant, anti-cancer, neuroprotective, hepatoprotective, and anti-diabetic activities can be related to the presence of compounds such as polysaccharides, lignans, phenolic glycosides, and saponins [102]; however, the molecular mechanisms should be elucidated. The less studied effects of flower consumption include anti-diarrheal, anti-depressant, and anti-spasmodic effects, a decrease in pain, protection against the development of gastric ulcers, an increase in the relative abundance of *Firmicutes* present in the gut microbiota, and the inhibition of enzymes involved in the aging process, among others potential benefits [103,104,105,106,107,108,109].

**Table 3:** Potential health benefits and risks associated with the intake of different edible flowers. The evidence supporting these effects is derived from in vitro studies or clinical observations.

Scientific Name	Potential Health Benefits and Risks		Sample Type	References
*Allium schoenoprasum* L.	Health benefits	Anti-proliferative	Phenolic compounds obtained from methanol extraction of the flower	[110]
	Risks	N.D		
*Borago officinalis* L.	Health benefits	Anti-bacterial	Aqueous, ethanol, and methanol extracts	[111,112]
		Antioxidant	Aqueous, ethanol and methanol extracts	[20,42,111,112,113]
		Anti-ulcer activity	Aqueous, methanol, and organic extracts	[103]
		Asthma symptoms reduction	Hydroalcoholic extract	[114]
		Hepatoprotective	Bioactive fractions derived from ethanol extract	[113]
		Pain reduction	Hydroalcoholic extract	[108]
	Risks	Cytotoxicity	Organic extract	[103]
		No information regarding toxic effects in humans	-	[51,115]
		Potential risks due to presence of alkaloids (1,2-unsaturated pyrrolizidine alkaloids)	-	[51]
*Bougainvillea glabra*	Health benefits	Anti-carcinogenic	Aqueous and methanol extract	[34,52]
		Anti-diabetic (by inhibition of α-glucosidase)	Aqueous and methanol extracts	[34,52]
		Anti-obesity (by inhibition of pancreatic lipase)	Aqueous extracts	[34]
		Antioxidant	Dry flowers; hydrophilic and methanol extracts	[20,34,38,45,52]
		Cardioprotective (preventing myocardial necrosis and oxidative stress)	Methanol extract	[116]
	Risks	No mortality of behavioral changes were observed	Methanol extract	[116]
		Non-toxic effects against normal cell lines	Ethanol extract of bracts	[117]
*Calendula officinalis* L.	Health benefits	Anti-bacterial (against *Klebsiella pneumonia*)	Methanol extract of flowers	[118]
		Hepatoprotective	Ethanol extract	[119]
		Neuroprotective (by increasing locomotor activity and attenuation of hippocampal dam age)	Methanol extract of flowers	[120]
		Anti-spasmodic	Aqueous-ethanol extract of flowers	[109]
	Risks	No information regarding toxic effects in humans	-	[51]
*Centaurea cyanus* L.	Health benefits	Antioxidant	Aqueous and methanol extract	[26,121]
		Anti-bacterial (against *Escerichia coli*, *Staphylococcus aureus* and *Listeria monocytogenes*)	Aqueous and ethyl acetate extracts of aerial parts	[122]
		Anti-hypertensive (by inhibition of ngiotensin I-converting enzyme—ACE)	Flower extract	[123]
		Antimicrobial (low effect)	Methanol extract of flower	[124]
	Risks	N.D		
*Chrysanthemum* spp.	Health benefits	Anti-carcinogenic	Methylene chloride fraction of *Chrysanthemum indicum* L.	[125]
		Anti-inflammatory (by suppressing TNF-α, IL-6 and COX-2)	Aqueous extract of *Chrysanthemum × morifolium*	[104]
		Antioxidant	Methanol extracts of *Chrysanthemum frutescens* and *Chrysanthemum parthenium*	[26]
		Anti-obesity (by inhibition of adipogenesis)	Aqueous extract of *Chrysanthemum morifolium* flowers	[126]
		Gut microbiota modulation (by increasing *Firmicutes* content)	Aqueous extract of *Chrysanthemum × morifolium*	[104]
		Hepatoprotective (by mitigation of liver injury)	Flavonoids (luteolin and luteoin 7-*O*-glucoside) extracted from petals of *Chrysanthemum × morifolium* and aqueous extract	[127,128]
		Neuroprotective	Flavonone glycosides derived from *Chrysanthemum morifolium* flowers extract	[129]
	Risks	Subclinical alterations in heart tissue; No clinical toxicity observed	Homogenates of *Chrysanthemum morifolium*	[130]
		No toxic effects observed	Ethanol extract of *Chrysanthemum morifolium* flowers	[131]
*Cichorium intybus* L.	Health benefits	Anti-diabetic (by α-amylase and α-glucosidase inhibition)	Ethanol extract of flowers	[66]
		Anti-diarrheal effect	Infusion of flowers	[132]
		Antioxidant	Ethanol extract of flowers	[66]
	Risks	N.D		
*Clitoria ternatea*	Health benefits	Antioxidant	Aqueous, ethanol and methanol extracts	[11,47,121,133,134]
		Antidiabetic (by pancreatic regeneration potential and anti-hyperglycemic effects)	Ethanol extract of flower and other aerial parts	[134]
		Anti-endocrine disrupting agent	Aqueous extract	[11]
		Anti-hemolysis	Aqueous extract	[133]
		Memory deficit attenuation	Ethanol extract of flower and other aerial parts	[135]
	Risks	Low toxicity (no mortality, but loss of mobility occurred)	Ethanol extract of flower and other aerial parts	[134]
		Lethargia, decreased locomotor activity and ptosis (dropping of upper eyelids)	Ethanol extract of flower and other aerial parts	[135]
*Cucurbita pepo* L.	Health benefits	Antidiabetic (by inhibition of α-glucosidase)	Ethanol extract of flowers	[136]
		Cholesterol reduction	Ethanol extract of flowers	[136]
	Risks	Presence of trypsin inhibitors was detected. No alkaloids, cyanogenic glycosides or hemolytic activity were identified	Sundried commercial flowers	[88]
*Hibiscus* L.	Health benefits	Anti-inflammatory	Aqueous extract and anthocyanin isolated from *Hibiscus sabdariffa* L.	[137,138]
		Anti-hypertensive	Infusion of dried calyces of *Hibiscus sabdariffa* L.	[139,140]
		Anti-obesity (by inhibition of adipogenesis)	Aqueous extract of *Hibiscus sabdariffa* L.	[141]
		Antioxidant	Aqueous extract of red flowers	[20]
	Risks	Diarrhea, hepatotoxicity, possible death	Aqueous and ethanol extracts of *Hibiscus sabdariffa* L.	[142]
		Toxic effects	*Hibiscus sabdariffa* Calyx extract	[143]
		Possible liver and heart injury when using for long periods	Methanol extract of red calyces of *Hibiscus sabdariffa* L.	[144]
		Interference with drugs (Acetaminophen)	Aqueous extract of red calyces of *Hibiscus sabdariffa* L.	[145]
*Jasminum sambac*	Health benefits	Ant carcinogenic	Methanol extract	[146]
		Antimicrobial (against *S. aureus*, *E. coli*, *Candida albicans*)	Ethanol extract	[147]
		Antioxidant (low effect)	Methanol extract	[148]
	Risk	No toxicity	Ethanol extract	[149]
*Lavandula angustifolia*	Health benefits	Anti-aging (by inhibition of acetylcholinesterase)	Methanol extract	[150]
		Anti-Hyperglycemic (by inhibition of α-amylase)	Methanol extract	[150]
		Anti-depressant	-	[151]
	Risks	Safe to use as a flavor agent	-	[152]
*Malva sylvestris* L.	Health benefits	Anti-bacterial (against *Bordetella bronchiseptica, Erwinia carotovora*, *S. aureus*, *Streptoccocus agalactiae*, and *Enterococcu*s *faecalis*). Bacteriostatic (against *S. aureus*)	Methanol extract	[153,154,155]
		Antidiabetic (by inhibition of α-amylase and α-glucosidase)	Ethanol extract	[66]
		Antifungal activity (modest) (against *Sclerotinia sclerotiorum*, *Candida kefyr*, *C. albicans*)	Methanol extract	[153]
		Antioxidant	Ethanol extract	[74,121,156]
		Skin elasticity increase	Aqueous extract	[157]
		Triglycerides reduction	Aqueous extract	[157]
	Risks	N.D		
*Matricaria chamomilla*	Health benefits	Anti-depressant (by increasing mobility)	Hydroalcoholic extract of flowers	[105]
		Anti-diabetic (by lowering glucose and protection of pancreatic islet cells)	Hydroalcoholic extract of aerial parts	[158]
		Antioxidant	Subcritical water extract (210 °C) of flowers	[159]
		Anti-spasmodic	Hydroalcoholic extract	[106]
		Anti-ulcerative colitis (by reducing inflammation, oxidative stress and immune response biomarkers)	Hydroalcoholic extract	[160]
		Cytotoxicity to malign cells	Subcritical water extract (115 °C) of flowers	[159]
		Memory improvement (by modulating cholinergic activity and neuroinflammation)	Hydroalcoholic extract of flowers	[161]
		Reduction in lung damage (by reduction in pulmonary fibrosis)	Hydroalcoholic extract of flowers	[162]
		Wound-curing (by increasing the production of growth factors)	Hydroalcoholic extract	[163]
	Risks	No signs of toxicity observed	Hydroalcoholic extract of flowers	[161]
*Nelumbo nucifera*	Health benefits	Anti-obesity (by inhibition of the differentiation of preadipocytes to adipocytes)	Methanol extract	[164]
		Hypolipidemic and hypoglycemic	Dry flowers	[165]
	Risks	Genotoxicity when reacting with nitrite (consumers should avoid any nitrite-containing food items)	Methanol extract	[166]
*Rhododendron arboreum*	Health benefits	Antioxidant	Ethanol extract	[69]
		Cardioprotective	Ethanol extract of petals	[167]
	Risks	Possible presence of grayanotoxins which can lead to intoxication. Authors state that *Rhododendron* plants are poisonous	*-*	[168]
		Rhododendron honey, flowers or medicinal preparations can lead to intoxication	*-*	[169]
		Toxic effects (convulsions, hypotension, paralysis, vomits)	All parts of rhododendron	[130]
*Rosa* spp.	Health benefits	Anti-aging (by Inhibition of skin aging-related enzymes)	Ethanol extract	[107]
		Anti-bacterial (against *Staphylococcus epidermidis*, *S. aureus*, *Bacillus subtilis*, *Micrococcus luteus*, *E. coli*, *K. pneumoniae*, *Pseudomonas aeruginosa*, *Proteus mirabilis*)	Aqueous and methanol extracts	[170]
		Anti-carcinogenic	Aqueous and methanol extracts	[170]
		Anti-diabetic (by Inhibition of α-glucosidase)	Methanol extract of flower	[171]
		Anti-inflammatory on skin tissues	Ethanol extract	[172]
		Antioxidant	Aqueous, ethanol and methanol extracts. Dry petals	[107,173,174]
		Anti-Parkinson’s and neuroprotection (by protection of nerve cells and improvement of motor symptoms and balance disorders)	Ethanol extract	[175]
	Risks	Low cytotoxicity on kidney epithelium; cytotoxic to blood leukocytes	Ethanol and methanol extract	[176]
*Tagetes erecta*	Health benefits	Anti-carcinogenic	Aqueous extract	[34]
		Anti-diabetic (by inhibition of α-glucosidase)	Aqueous extract	[34]
		Anti-inflammatory	Hydroalcoholic extract	[177]
		Anti-obesity (by Inhibition of pancreatic lipase)	Aqueous extract	[34]
		Antioxidant (strong effect)	Ethanol, hydrophilic, and hydroethanolic extracts	[21,34,178]
		Anti-parasite	Aqueous extract	[179]
	Risks	No lethality or toxic effects	Aqueous extract	[179]
*Taraxacum officinale* Weber	Health benefits	Anti-angiogenic	Ethanol extract of aerial parts	[180]
		Anti-bacterial (against *Helicobacter pylori*)	Aqueous and ethanol extracts	[181]
		Anti-carcinogenic (against human colon colorectal adenocarcinoma)	Aqueous and ethyl acetate extracts	[182]
		Anti-diabetic (by serum glucose reduction)	Aqueous and ethanol extracts	[183]
		Anti-inflammatory	Aqueous and ethanol extracts	[180,181]
		Anti-nociceptive	Ethanol extracts	[180]
		Antioxidant	Aqueous, ethanol, and ethyl acetate extracts	[182,184]
		Gastroprotective	Ethanol extract	[181]
		LDL-cholesterol and triglycerides reduction, HDL-cholesterol increase	Aqueous extract	[183]
	Risks	Allergic reaction.to pollen	-	[185]
		No lethality	-	[186]
*Tropaeolum majus* L.	Health benefits	Antiarthritic (low effect)	Methanol extracts of aerial parts	[187]
		Antimicrobial (against *Bacillus cereus*, *Pseudomonas* spp., *Acinetobacter* spp., *Staphylococcus* spp., *Enterococcus* spp., and *Klebsiella* spp.)	Methanol extract of flowers	[188]
		Anti-obesity (anti-adipogenic effect and (by inhibition of pancreatic lipase)	Ethanol and methanol extracts	[187,189]
		Antioxidant	Aqueous extract of flower	[20]
		Hepatoprotective (by preservation of hepatic tissues)	Methanol extract of flowers and leaves	[190]
	Risks	High doses (>39.5 g) can exceed the daily intake of erucic acid	-	[8,51]
		Mortality	Aqueous, hydro-ethanol, and methanol extract of flowers and leaves	[190]
*Tulipa gesneriana*	Health benefits	Antimicrobial (against *S. aureus, Enterobacter cloasea, Salmonella typhimurium*, *E. coli*, *Yersinia enterocolitica*, *L*. *monocytogenes*, *B.cereus*, and *B. subtilis)*	Anthocyanin-based extracts of red tulips	[42]
	Risks	Only petals are edible	-	[28]
		Red tulip consume can be questioned	-	[42]
		Yellow and clared red tulip flowers can be toxic	-	[191]
*Viola tricolor*	Health benefits	Antioxidant	Fresh flowers irradiated	[192]
	Risks	Possible health issues for individuals with sensitivity to salicylic acid due to Methyl salicylate presence	-	[51]
*Viola × wittrockiana*	Health benefits	Antioxidant	Methanol extract	[42,193]
		Neuroprotection (by inhibition of neurodegenerative enzymes)	Ethanol extract of flower	[194]
	Risks	N.D		

N.D: no data; b.w: body weight.

As can be seen in Table 3, studies on the health properties of raw flowers are scarce. Most of the research on this topic has been carried out using aqueous, ethanolic, and methanolic extracts derived from petals or other aerial parts of the plant. The proven properties of ethanolic extracts of edible flowers include benefits for the liver, heart, and brain [113,119,135,167,194].

Furthermore, infusions made using some edible flowers may be an interesting alternative for patients that seek to reduce body weight, since the anti-obesity properties of aqueous extracts of *Bougainvillea glabra*, *Chrysanthemum morifolium*, *Hibiscus sabdariffa* L., and *Tagetes erecta* have shown effects in inhibiting adipogenesis and enzymes involved in lipid digestion [34,126,141]. In addition, the consumption of aqueous extracts of edible flowers like *Bougainvillea glabra*, *Tagetes erecta*, and *Taraxacum officinale* has been shown to reduce the incidence of diabetes [34,183].

Many of the commonly studied flowers show properties against the proliferation of several types of microorganisms. Among edible flowers, *Borago officinalis* L., *Calendula officinalis* L., *Centaurea cyanus* L., *Malva sylvestris* L., *Rosa* spp., *Taraxacum officinale* Weber, *Tropaeolum majus* L., and *Tulipa gesneriana* showed the potential to inhibit the growth of different species of *Bacillus*, *Staphylococcus*, *Escherichia*, *Enterococcus*, and *Salmonella*, as well as some fungal species [42,111,118,122,153,154,155,170,181,188].

**Figure 2:**
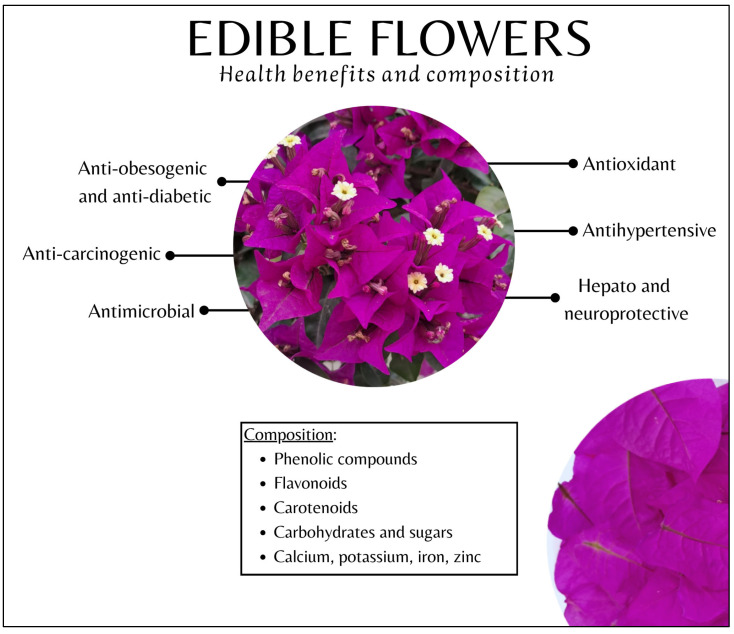
Main health benefits and nutritional composition of edible flowers.

Figure 2 summarizes the most relevant benefits for human health related to edible flower intake, as well as their main nutritional components.

Despite the benefits mentioned, many edible flowers are still under study to understand how best to consume them and maximize health benefits while avoiding risks. In this sense, there is still a long way to go before they are included in the list of foods that can be consumed. Different toxicological studies on cells and animal models have shown that most edible flower extracts are probably safe and non-toxic (Table 3). Nevertheless, some investigations have found possible toxic effects associated with extracts from *Borago officinalis* L., *Chrysanthemum* spp., *Clitoria ternatea*, *Rhododendron arboretum*, and *Rosa* spp. and other species [51,130,135,168,169,176]. A detailed discussion on the general safety aspects regarding the consumption of edible flowers can be found in Section 6.

## 5. Safety Aspects of Edible Flower Consumption

Several authors highlighted safety issues related to the consumption of edible flowers [1,7,8,169,195]. A review article written by Egebjerg et al. [51] summarizes some of the edible flowers with potentially toxic substances. Although there is insufficient information to determine the toxicity of some of the species studied, these authors have indicated the presence of possibly toxic compounds in *Borago officinalis* L., *Tropaeolum majus* L., *Viola tricolor* L., *Achillea millefolium* L., *Echium vulgare* L., and *Syringa vulgaris* L. In a survey carried out by Guiné et al. [60], among 559 adults from three countries, less than half of them were aware of the risk of consuming edible flowers, with most individuals concerned about the presence of pesticides. Studies regarding the risk of edible flower consumption are summarized in Table 3.

Edible flowers may contain toxic or poisonous substances, including alkaloids, cyanogenic glucosides, oxalic acid, saponins, terpenes, and erucic acid [7,8]. Alkaloids can exert stimulant or psychotropic effects. These substances are usually bitter and act as a defense mechanism for the plant [3,7,8,41]. In addition, some flower species contain anti-nutritional factors like saponins, trypsin inhibitory enzymes, and hemagglutinating activity [7] that may interfere with the absorption or metabolism of certain nutrients. In addition, there is currently a lack of scientific information on the use and safe doses of different flowers [1], and as previously stated, pathogenic microorganisms can be found in edible flowers. 

Besides this, confusion can occur between toxic and non-toxic species that are visually similar. Some flowers of the same family can be toxic or edible, such as the flowers of *Pisum sativum* (common pea, edible) and *Lathyrus odoratus* (sweet pea, toxic). In this case, both flowers can have similar colors and some morphological characteristics [3,51].

Allergies to edible flowers can occur in susceptible individuals. Some parts of the flowers (specifically the reproductive organs) can induce allergies [3] and pollen found in flowers may cause various reactions in people allergic to them. Finally, a possible interaction between active principles of flowers and drugs could occur, as in the case documented by Kolawole et al. [145] in samples of *Hibiscus sabdariffa.*

Some flowers mentioned in this article can be helpful to preventing or delaying the onset of diverse health conditions, as well as improving some of their signs and symptoms. However, it is important to consider whether these flowers can be used safely as part of ‘alternative medicine’ or not. According to González-Castejón et al. [36], edible flowers should not be considered medicinal products, especially for individuals with severe health disorders. In cases where a plant is consumed in its totality, such as dandelion, the information about the safe amount of leaves or roots is already available [36]. According to a review carried out by González-Castejón et al. [36], the amounts usually consumed are below 50 g/day of fresh roots or leaves, and less than 10 g/day of dried roots or leaves. However, information regarding the safe intake of the flowers is not so widespread. There are many missing points regarding the available information of the safety of these kinds of flowers, including their toxicological profile and adequate taxonomy identification in order to determine the species that are genuinely edible [7,196]. In this sense, public and reliable access to information on safe edible flower species and their optimal doses would be an important way to take care of consumers [196]. Moreover, it would be important to have a legal regulation label regarding edible flowers and a government commitment to spread knowledge on this topic. As with other foods that are considered “unconventional”, it is important for consumers to be cautious with the edible flowers available, knowing their origin, acquiring them through reliable stores, and evaluating individual tolerance over time.

## 6. Microbiological Aspects of Edible Flowers

Despite their health benefits, edible flowers are highly perishable and can cause significant safety concerns. Due to their chemical composition and high water content, fresh flowers are exposed to microbial spoilage during storage. Some flowers can be toxic or cause allergic reactions if consumed raw or improperly processed. Furthermore, some blooms are harmful especially due to the presence of pathogens that contaminate them at any stage of their lifecycle (soil, water, post-harvesting, handling, and packaging), as highlighted by the Rapid Alert System for Food and Feed (RASFF), even more so because edible flowers are usually consumed fresh without prior heat treatment [197].

Many of the species isolated and identified from edible flower samples are potentially pathogenic for humans, including *Salmonella* spp., *Enterobacter* spp., *Bacillus* spp., and *S. aureus* that raise potential food safety concerns, emphasizing the importance of proper cultivation and processing practices to ensure consumer safety [198]. Molds and yeasts are also considered undesirable microorganisms, as they compromise both the nutritional and sensory characteristics of flowers, posing risks to product quality and consumer health. Fungi can produce volatile compounds that cause off-flavors and texture alterations, negatively affecting the sensory appeal of edible flowers. Furthermore, they accelerate aging and spoilage during storage, degrading plant tissues through enzymatic activity, thereby reducing shelf-life and marketability [199]. Some molds, such as *Aspergillus*, *Penicillium*, *Alternaria*, or *Fusarium*, can produce mycotoxins, while others, such as *Cladosporium* and *Alternaria*, can cause allergies [200,201,202]. Table 4 summarizes the main microorganisms identified in different species of edible flowers by culture-dependent and independent methods.

Edible flowers, such as *Centaurea cyanus L*., *Tagetes erecta*, and *Azadirachta indica* (Margosa flower), have been reported to be contaminated by *Salmonella* spp. [204]. *Tagetes erecta* was also found to be contaminated by *S. aureus,* different yeasts (1.30–2.08 log CFU/g), and molds (2.30–4.76 log CFU/g) despite a decontamination treatment made with ozone [205]. In fact, Wilczyńska A. et al. [205] studied this process as a promising method for microbiological decontamination, particularly for edible plants or flowers. Ozone (O_3_) is a powerful oxidant with broad-spectrum antimicrobial properties, capable of eliminating bacteria, fungi, and other microorganisms [220]. However, its effectiveness is influenced by factors such as ozone concentration, exposure time, and the presence of organic matter that can reduce its efficiency.

Shalini et al. [117] focused on the microbiological component of *Bougainvillea glabra* bracts, assessing their safety as an edible flower. Total mesophilic bacteria and *Eumycetes* were below the allowable limit, indicating that the flowers were safe for consumption and not microbially contaminated. Lara-Cortés et al. [211] conducted a study to identify enteric bacteria associated with *Dahlia* spp., focusing on morphological, biochemical, and molecular methods. The research aimed to explore the pathogens occurring on these edible flowers that have a historical significance in food consumption. Preliminary results highlighted the potential presence of *E. coli*, *Salmonella* spp., and *Enterobacter cancerogenus*. The main isolate identified in the study was *Pantoea vagans*, representing the first report of this species isolated from *Dahlia* spp. flowers. *P. vagans* is isolated from a variety of geographic locations and ecological sources, including soil, water, seeds, plants, and people. To ensure the microbiological safety of their products, floriculturists must adhere to appropriate hygiene standards, making use of antimicrobial substitutes that are safe for consumers and do not harm the phytosanitary condition of the flower.

Fürnkranz et al. [208] examined the variety of endophytic microbial communities from *Cucurbita pepo* L. flowers. In detail, several pathogenic species were identified, specifically *B. flexus*, *B. gibsonii*, *B. indicus*, *B. firmus*, *B. subtilis*, *P. viridiflava*, and *P. syringae.* The flowers were also examined for fungal contamination revealing the presence of ascomycetes such as *P. cucumerina*, *P. herbarum*, *Pleosporaceae* spp., *Capnobotryella* spp., and *Oidiodendron* spp.

Baruzzi et al. [210] further studied the microbial dynamics of *Cucurbita pepo L*. stored at low temperature (4 °C). Their study found that the microbial load on the pistils was consistently higher than that on the petals, with total aerobic mesophilic bacteria showing significant differences between these parts. The greater availability of nutrients in the pistils probably contributed to this higher microbial proliferation.

Wilczynska et al. [198] carried out a study on the microbial contamination of several edible flowers, including *Tropaeolum majus* L., *Calendula officinalis* L., *Dianthus caryophyllus* L., *Bellis* (daisy), and *Hemerocallis* L. (daylili). They found that all samples were contaminated with *S. aureus*, with counts ranging from 1.24 to 2.94 log CFU/g. In addition, yeasts and molds were detected in all samples, with contamination levels depending on the type of flower, ranging from 3.72 to 5.85 log CFU/g. *E. coli* was only found in *Tropaeolum majus* L. and *Bellis* samples. Furthermore, daisy flowers exhibited the highest levels of *S. aureus,* while daisy flowers showed the lowest levels of contamination [205]. In another study, Wetzel et al. [217] found pathogens like *Enterobacter* spp., *Bacillus* spp., *P. aeruginosa, S. enterica* subsp. *enterica* serovar Typhimurium.

Lee et al. [203] investigated the food safety of *Chrysanthemum morifolium* RAMAT*, Jasminum sambac* L., *Matricaria recutita* L., *Viola × wittrockiana, Acacia decurrens* (acacia), *Pueraria lobata Ohwi* (kudzu), *Magnolia kobus* A. P. DC. (magnoilia), and *Prunus serrulata var. spontanea* (prunus), *Jasminum sambac* L., *Matricaria recutita* L., *Acacia decurrens,* and *Magnolia kobus A. P. DC* contained aerobic bacteria (2.7–4.48 log CFU/g), *Listeria* spp. (2–2.48 log CFU/g), and *S. aureus (*2–2.3 log CFU/g*)*, while *Viola × wittrockiana* and *Prunus serrulata var. spontanea* contained only aerobic bacteria (2–3.35 log CFU/g) and *Listeria* spp. (1.7–2 log CFU/g). *Chrysanthemum morifolium* RAMAT was the only dried flower showing only aerobic bacterial load (2.6 log CFU/g) without the presence of pathogenic organisms. This highlighted the need to implement strict quality controls in the production of edible dried flowers to ensure food safety, adopting sustainable agricultural practices, regular monitoring of products, and consumer education on the potential risks associated with consuming contaminated dried flowers.

Seidler-Lozykowska et al. [213] examined the organic and conventional cultivation of *Lavandula angustifolia* Mill. in Poland. Various parameters were compared, including the level of microbiological contamination. Depending on the origin of the flowers, the microbiological contamination of the raw materials varied greatly. The organic flowers had the highest levels of aerobic bacteria, yeasts, and molds, compared to conventional flowers. The flowers from the organic production had the highest level of *Enterobacteriaceae*. However, all raw materials examined were below the acceptable contamination levels established by the European Pharmacopoeia standard (2010) [221]. After six months of storage, microbial contamination decreased at varying rates, probably for two main reasons: the fact that bacterial species are more or less susceptible to drying, and the presence of plant compounds with antimicrobial properties, such as essential oils, anthocyanins, and tannins which can affect microbial survival.

Wetzel et al. [217] also examined the microbial biota of edible flowers, specifically *Pelargonium hortorum* and *Viola tricolor* cultivated in organic conditions. Their study revealed the presence of various pathogens, including *Enterobacter* spp., *Bacillus* spp., *P. aeruginosa* and *S. enterica* subsp. *enterica* serovar Typhimurium, with a significant number of isolates belonging to the *Enterobacteriaceae* family. Three main factors can contribute to the microbial presence in these flowers: temperature, selective medium, and native plant-bacteria interactions. Most *Enterobacteriaceae* species arise from fecal contamination during pre- or post-harvest handling processes. The presence of *Enterobacteriaceae* could indicate poor hygienic practices during handling and processing. Furthermore, the packaging of edible flowers can exacerbate the risks of microbial contamination.

Sasu et al. [209] discovered that cucumber beetles could lead to microbial contamination in edible flowers. These beetles feed on cucurbitacin in the flowers’ anthers, leaving behind excreta that exposes the petals to pathogens such as *Erwinia tracheilphila*. Once inside the plant, the bacteria multiply in the xylem, producing an exopolysaccharide matrix that stops the water flow and causes wilting symptoms, which typically manifest 7–15 days after infection.

In addition to bacteria, edible flowers can also be contaminated by fungi. This is the case of *Hibiscus rosa-sinensis* for which a fungal disease caused by the fungus *Choanephora infundibulifera* has been reported in Korea in 2013 [212] and previously in other countries, including Japan, Myanmar, and the United States. The initial symptoms of the disease manifested as red-violet spots at the tips of the flowers, which later developed into reddish-brown, water-soaked lesions, leading to rapid decay of the affected flowers. Furthermore, some authors found that petunia can be contaminated by *Phytophthora cryptogea,* a pathogen responsible for the rapid decline of petunias [218] and *Botrytis cinerea* [219], the most common, devastating and pathogenic fungus that causes gray mold in greenhouse-grown petunias.

Ruiz Rodríguez et al. [222] carried out a microbial analysis of various edible flowers in Northern Argentina, including papaya flowers (*Carica papaya* L.). Their findings revealed the presence of total microbial counts, coliforms, and lactic acid bacteria.

Carpena et al. [216] carried out a comprehensive study assessing the chemical and microbiological risks associated with the consumption of wild edible plants (WEPs) and flowers, specifically focusing on *Ocimum basilicum* (basil), *Origanum vulgare* (oregano), *Salvia rosmarinus* (rosemary), and *Thymus vulgaris* (thyme). The research revealed significant contamination by various pathogens, with *Salmonella* spp. being the most frequently reported pathogen, followed by *B. cereus* and *E. coli. Ocimum basilicum* was identified as the most contaminated herb, exhibiting high microbial loads. Yeasts and molds were present only in *Ocimum basilicum* with counts ranging from 4.92 to 5.35 log CFU/g; aerobic mesophilic bacteria were found in *Ocimum basilicum*, with counts oscillating from 5.00 to 6.95 log CFU/g. Mesophilic bacteria counts ranging from <10 to 1.2 × 10^7^ CFU/g were found in *Origanum vulgare*; *E. coli* and *Enterobacteriaceae* were present at counts of 1.4–6.5 log CFU/g and 6.47 log CFU/g, respectively. *B. cereus* was present only in *Salvia rosmarinus* with counts ranging from 1 to 5 × 10^6^ CFU/g, while *L. monocytogenes* and *C. perfringens* were found only in *Salvia rosmarinus* and *Thymus vulgaris* with a count of 1.5 × 10^3^–7.9 × 10^3^ CFU/g and 0.8 × 10^3^–2.5 × 10^3^ CFU/g, respectively. In a previous study, Wetzel et al. [215] compared organic and conventional *Ocimum basilicum*, revealing a higher microbial diversity associated with organically grown samples, due to the absence of chemical fertilizers, which can expose plants to multiple sources of environmental contamination, such as animal manure. The results demonstrated the presence of *Enterobacter* spp., *Bacillus* spp., *P. aeruginosa*, *S. enterica* subsp. *enterica* serovar Typhimurium, *E. raffinosus*, *Erwinia* spp. and *K. singaporensis*. In particular, a large number of *Enterobacter* spp. was found in all the samples, indicative of indigenous microbial flora associated with WEPs and edible flowers. This aspect has also been observed in other edible flowers, such as *Pelargonium hortorum.*

### Methods to Reduce Microbiological Load in Edible Flowers

Several techniques, including dehydration, drying, vacuum, microwave, and hybrid methods, freezing, and high-pressure processing (HPP) are currently being explored to enhance the microbial safety of edible flowers, extend their shelf-life, and preserve their bioactive compounds. Wilczyńska et al. [205] examined the effects of different packaging methods on microbial quality during cold storage of *Tropaeolum majus* L., *Calendula officinalis* L. and daisy (*Bellis*). Flowers stored in vacuum-sealed PA/PE bags and PET cartons showed minor visual alterations after three days of refrigeration. *Tropaeolum majus* L. and *Calendula officinalis* L. showed minimal changes compared to daisies, which exhibited more wilting. After three days in refrigeration, *Tropaeolum majus* L. was the only flower type still contaminated with *E. coli*. *Calendula officinalis* L. flowers had a *S. aureus* count of 1.89 log CFU/g, while daisies had a substantially higher count of 2.72 log CFU/g, but *S. aureus* was present in all of the samples. Yeasts and molds counts remained relatively stable across the different flower types during refrigeration, with only a slight decrease observed in *Tropaeolum majus* L. The findings highlight the importance of proper handling and storage conditions to mitigate the risk of microbial contamination in edible flowers. Packaging plays a critical role in preventing desiccation, maintaining the delicate structure of flowers and reducing their exposure to microbial decontamination and other pollutants [223]. Even with precautions, some bacteria and fungi can still develop and damage edible flowers due to their unpredictable nature [199]. One of the simplest and most straightforward techniques for preserving edible flowers is low-temperature storage. Depending on the species, edible flowers might be satisfactorily preserved at 4 °C for 7–14 days [224]. Hence, combining low-temperature storage with appropriate packaging is crucial to preserve the microbial integrity of edible flowers over time.

In a study conducted by Fernandes et al. [206], the effect of freezing on the microbial quality of edible flowers, specifically *Viola tricolor* together with *Taraxacum officinale*, *Borago officinalis*, and *K. blossfeldiana* (Yellow kalanchoe) was assessed for the first time. The research focused on how the storage at −18 °C for three months influenced the microbial counts, examining both fresh flowers and those frozen in ice cubes. Fresh flowers had different microbial levels, with *Borago officinalis* showing the lowest counts and *K. blossfeldiana* the highest. The average number of molds and yeasts in fresh edible flowers was approximately 2 log CFU/g. Although freezing led to a reduction or maintenance of microbial growth, *Taraxacum officinale* showed an increase in molds and aerobic mesophilic bacteria post-freezing. In addition, the fresh flowers had levels of *E. coli* and total coliforms below 1 log CFU/g, indicating good hygiene practices during manufacturing. Flowers frozen in ice cubes had lower microbial counts than those frozen individually, likely due to better protection from external contaminations. For optimal safety, thawing of flowers should be carried out in the refrigerator rather than at room temperature, and refreezing should be avoided. Fernandes et al. [99] also explored the shelf life of four edible flowers: *Borago officinalis*, *Centaurea cyanus*, *Viola wittrockiana*, and *Camellia japonica* (rose camelias) focusing on the effects of different high hydrostatic pressure (HHP) treatments. Total mesophilic bacteria, yeasts, molds, and total coliforms were analyzed in both untreated and HHP-treated flowers. Untreated flowers showed significantly higher microbial loads (total aerobic mesophilic and molds) with varying post-harvest behaviors: *Borago officinalis* deteriorated rapidly within 1 day; *Centaurea cyanus* had the longest shelf-life at 12 days; *Viola wittrockiana* and *Camellia japonica* had intermediate shelf life of about 6 days.

## 7. Conclusions

Edible flowers have been consumed since ancient times in several dishes including beverages, salads, soups, and desserts, and they are becoming an important ingredient in modern cuisine and in food technology applications due to their sensory characteristics such as particular taste, aroma, and texture. In addition, health benefits associated with edible flowers are many and diverse, and include antioxidant, anti-obesogenic, anti-diabetic, and antimicrobial properties, as well as neuro- and hepatoprotective functions.

Despite the many advantages of edible flower consumption, there is still a lack of information about their safe intake, the identification of species, and the risks and contaminants associated with their ingestion. In this context, empirical and ancient knowledge may still be highly relevant. Due to their delicate nature, edible flowers are susceptible to contamination by different undesirable microorganisms, necessitating effective microbial control measures. The most common types of microbial contamination in flowers are usually caused by bacteria and fungi. *Bacillus* spp., *Enterobacte*r spp., *Salmonella* spp., and *Staphylococcus aureus* are among the more frequent bacteria in edible flowers. In this sense, several methods to reduce the microbiological load are being evaluated. Proper agricultural practice and hygienic handling are essential to ensure that the product is harvested with the lowest possible load. On the other hand, appropriate storage conditions and packaging are useful to minimize microbial contamination, ensure the safe consumption of edible flowers, prolong their shelf-life, and preserve their bioactive compounds.

Looking ahead, it is important to assess how best to meet current and future demand for edible flowers. As the consumption of the flowers increases, it will become increasingly important to ensure their safety. Beyond the scientific literature currently available and discussed in the present review, there is still a long way to go in promoting the consumption of flowers as food and ensuring their microbiological safety remains imperative. Public access to accurate, consistent, and trustworthy information on the safety, proper use, and handling of edible flowers will be key to promoting responsible consumption.

## Figures and Tables

**Table 4:** Microbiological aspects of edible flowers by culture-dependent and -independent methods.

Scientific Name	Microorganisms Present in Flower	Origins of Flowers	References
*Acacia decurrens*	Aerobic bacteria, *S. aureus*, *Listeria* spp.	Republic of Korea	[203]
*Azadirachta indica*	*Salmonella* spp.	Thailand	[204]
*Bellis*	*S. aureus*, yeasts and molds, *E. coli*	Poland	[198,205]
*Borago officinalis*	Aerobic Mesophilic bacteria, psychotropic bacteria*,* yeasts and molds, total coliforms	Portugal	[99,206]
*Bougainvillea glabra*	Aerobic bacteria, yeasts and molds; *Fusarium oxysporum*	Malesia	[117,207]
*Calendula officinalis*	*S. aureus*, yeasts and molds, *E. coli*	Poland	[198,203]
*Camellia japonica*	Aerobic Mesophilic bacteria, yeasts and molds, total coliforms, psychotropic bacteria	Portugal	[99]
*Centaurea cyanus* L.	*Salmonella* spp. Aerobic Mesophilic bacteria, yeasts and molds, total coliforms, psychotropic bacteria	Albania, Portugal	[99,204]
*Chrysanthemum morifolium*	*Aerobic bacteria*	Republic of Korea	[203]
*Cucurbita pepo L.*	*Bacillus flexus*, *Bacillus gibsonii*, *Bacillus indicus*, *Bacillus firmus*, *B. subtilis*, *Pseudomonas viridiflava*, *Pseudomonas syringae*, *Plectosphaerella cucumerina*, *Phoma herbarum*, *Oidiodendron* spp., *Capnobotryella* spp., *Pleosporaceae* spp.; *Erwinia tracheiphila*; Mesophilic aerobic bacteria, yeasts and molds, *Acinetobacter* spp., *Staphylococcus* spp., *Arthrobacter* spp., *Serratia* *marcescens*, *Enterobacter* spp., *Pantoea* spp., *Weissella* spp., *Klebsiella* spp., *Erwinia* spp., *Pseudoclavibacter* spp., *Bacillus* spp., *Pseudomonas* spp. (cold storage)	Austria, USA, Italy	[208,209,210]
*Dahlia* spp.	*Pantoea vagans*	Mexico	[211]
*Dianthus caryophyllus* L.	*S. aureus*, yeasts and molds, *E. coli*	Poland	[198]
*Hemerocallis* L.	*S. aureus*, yeasts and molds, *E. coli*	Poland	[198]
*Hibiscus rosa-sinensis* L.	*C hoanephora infundibulifera*	Republic of Korea	[212]
*Jasminum sambac* L.	Aerobic bacteria, *S. aureus*, *Listeria* spp.	China	[203]
*Kalanchoe blossfeldiana*	Aerobic Mesophilic bacteria, psychotropic bacteria, Yeasts	Portugal	[206]
*Lavandula angustifolia*	Aerobic bacteria, yeasts and molds, *Enterobacteriaceae*	Poland	[213]
*Magnolia kobus A. P. DC.*	Aerobic bacteria*, S. aureus*, *Listeria* spp.	Republic of Korea	[203]
*Matricaria recutita* L.	Aerobic bacteria, *S. aureus*, *Listeria* spp.	Republic of Korea	[203]
*Nelumbo Nucifera*	*S. marcescens; Erwinia* spp., *Sphingomonas* spp., *Dickeya* spp., *Escherichia*-*Shigella* spp., *Pantoea* spp., *Serratia* spp., *Raoultella* spp.	China, India	[214,215]
*Ocimum basilicum*	Aerobic bacteria, Mesophilic bacteria, yeasts and molds; *Enterobacter* spp., *Bacillus pumilus*, *Bacillus stratosphericus*, *P. aeruginosa*, *Salmonella enterica* subsp. *enterica* serovar Typhimurium, *Erwinia* spp., *Klebsiella singaporensis*, *Enterococcus raffinosus*	Spain, Poland, USA	[216,217]
*Origanum vulgare*	Mesophilic bacteria, *E. coli*, Enterobacteriaceae	Spain, Poland	[216]
*Pelargonium hortorum*	*Enterobacter* spp., *Bacillus* spp., *P. aeruginosa, S. enterica* subsp. *enterica* serovar Typhimurium	USA	[217]
*Petunia hybrida*	*Phytophthora infestans*, *Phytophthora cryptogea*, *Botrytis cinerea*, *Phytophthora parasitica* Dast. (syn. *Phytophthora nicotianae* Breda de Haan.)	USA, Africa, Chile, Romania	[218,219]
*Prunus serrulata var. spontanea*	Aerobic bacteria, *Listeria* spp.	Republic of Korea	[203]
*Pueraria lobata Ohwi*	Aerobic bacteria, *S. aureus*, *Listeria* spp.	Republic of Korea	[203]
*Salvia rosmarinus*	Aerobic Mesophilic bacteria, *B. cereus*, *L. monocytogenes*, *Clostridium perfringens*	Spain, Poland	[216]
*Tagetes erecta*	*S. aureus*, yeasts and molds*, Salmonella* spp.	Poland, Egypt	[204,205]
*Taraxacum officinale*	Yeasts and molds, Aerobic Mesophilic bacteria, psychotropic bacteria	Portugal	[206]
*Thymus vulgaris*	*L. monocytogenes*, *C. perfringens*, Mesophilic bacteria	Spain, Poland	[216]
*Tropaeolum majus* L.	*S. aureus,* yeasts and molds, *E. coli* *Enterobacter* spp., *Bacillus* spp., *P. aeruginosa*, *S. enterica* subsp. *enterica* serovar Typhimurium	Poland, USA	[198,217]
*Viola tricolor* L.	*Enterobacter* spp., *Bacillus* spp., *P. aeruginosa*, *S. enterica* subsp. *enterica* serovar Typhimurium, psychotropic bacteria	USA, Portugal	[206,217]
*Viola × wittrockiana* White/violet	Aerobic Mesophilic bacteria, *Listeria* spp., *E. coli*, *S. aureus*, yeasts and molds, total coliforms, psychotropic bacteria	Republic of Korea, Poland, Portugal	[99,203,205]

## Data Availability

No new data were created or analyzed in this study. Data sharing is not applicable to this article.

## Abbreviations

The following abbreviations are used in this manuscript:

b.w	Body weight
CAR	Carotenoids
CarboH	Carbohydrates
CF	Crude fiber
CFU	Colony forming unit
d.w	Dry weight
FDA	Food and Drug Administration
f.w	Fresh weight
N.D	No data
RASFF	Rapid Alert System for Food and Feed
TDF	Total dietary fiber
TF	Total flavonoids
TPC	Total polyphenol content

## References

[1] Kumar S., Uttam A., Sharma S., Kumar V. (2025). Edible Vegetable Flowers: Next Generation Sustainable Super Foods, Therapeutic Role, Processing and Improvement Approaches. Int. J. Gastron. Food Sci..

[2] Matyjaszczyk E., Śmiechowska M. (2019). Edible Flowers. Benefits and Risks Pertaining to Their Consumption. Trends Food Sci. Technol..

[3] Nicolau A.I., Gostin A.I. (2016). Safety of Edible Flowers. Regulating Safety of Traditional and Ethnic Foods.

[4] Chitrakar B., Zhang M., Bhandari B. (2019). Edible Flowers with the Common Name “Marigold”: Their Therapeutic Values and Processing. Trends Food Sci. Technol..

[5] Acikgoz F.E. (2017). Edible Flowers. J. Exp. Agric. Int..

[6] Rodrigues H., Spence C. (2023). Looking to the Future, by Studying the History of Edible Flowers. Int. J. Gastron. Food Sci..

[7] Benvenuti S., Mazzoncini M. (2021). The Biodiversity of Edible Flowers: Discovering New Tastes and New Health Benefits. Front. Plant Sci..

[8] Jadhav H.B., Badwaik L.S., Annapure U., Casanova F., Alaskar K. (2023). A Review on the Journey of Edible Flowers from Farm to Consumer’s Plate. Appl. Food Res..

[9] Bieżanowska-Kopeć R., Ambroszczyk A.M., Piątkowska E., Leszczyńska T. (2022). Nutritional Value and Antioxidant Activity of Fresh Pumpkin Flowers (*Cucurbita* sp.) Grown in Poland. Appl. Sci..

[10] Fernandes L., Pereira J.A., Saraiva J.A., Ramalhosa E., Casal S. (2019). Phytochemical Characterization of *Borago officinalis* L. and *Centaurea cyanus* L. During Flower Development. Food Res. Int..

[11] Goh S.E., Kwong P.J., Ng C.L., Ng W.J., Ee K.Y. (2021). Antioxidant-rich *Clitoria ternatea* L. flower and its benefits in improving murine reproductive performance. Food Sci. Technol..

[12] Chen N.H., Wei S. (2017). Factors influencing consumers’ attitudes towards the consumption of edible flowers. Food Qual. Prefer..

[13] Pires T.C., Dias M.I., Barros L., Barreira J.C., Santos-Buelga C., Ferreira I.C. (2018). Incorporation of natural colorants obtained from edible flowers in yogurts. LWT.

[14] Shiau S.Y., Yu Y., Li J., Huang W., Feng H. (2023). Phytochemical-rich colored noodles fortified with an aqueous extract of *Clitoria ternatea* flowers. Foods.

[15] Rashid S., Tong W.Y., Leong C.R., Abdul Ghazali N.M., Taher M.A., Ahmad N., Teo S.H. (2021). Anthocyanin microcapsule from *Clitoria ternatea*: Potential bio-preservative and blue colorant for baked food products. Arab. J. Sci. Eng..

[16] Qiu L., Zhang M., Mujumdar A.S., Chang L. (2021). Effect of edible rose (*Rosa rugosa* cv. *Plena*) flower extract addition on the physicochemical, rheological, functional and sensory properties of set-type yogurt. Food Biosci..

[17] Shirodkar S.M., Multisona R.R., Gramza-Michalowska A. (2023). The potential for the implementation of pea flower (*Clitoria ternatea*) health properties in food matrix. Appl. Sci..

[18] Li A.N., Li S., Li H.B., Xu D.P., Xu X.R., Chen F. (2014). Total phenolic contents and antioxidant capacities of 51 edible and wild flowers. J. Funct. Foods.

[19] Grzelczyk J., Drożdżyński P., Budryn G., Czarnecki A., Paprocka Z., Gałązka-Czarnecka I. (2025). High-fiber cookies with bamboo flour and edible flowers: Evaluation of structural properties, phenolic content, antioxidant activity and nutritional value. LWT.

[20] Conforti P.A., Patrignani M. (2025). Antioxidant activity from non-conventional beverage plant sources in Argentina. Beverage Plant Res..

[21] Navarro-González I., González-Barrio R., García-Valverde V., Bautista-Ortín A.B., Periago M.J. (2014). Nutritional composition and antioxidant capacity in edible flowers: Characterisation of phenolic compounds by HPLC-DAD-ESI/MSn. Int. J. Mol. Sci..

[22] Lu B., Li M., Yin R. (2016). Phytochemical Content, Health Benefits, and Toxicology of Common Edible Flowers: A Review (2000–2015). Crit. Rev. Food Sci. Nutr..

[23] Prabawati N.B., Oktavirina V., Palma M., Setyaningsih W. (2021). Edible flowers: Antioxidant compounds and their functional properties. Horticulturae.

[24] D’Antuono L.F., Manco M.A. (2013). Preliminary sensory evaluation of edible flowers from wild *Allium* species. J. Sci. Food Agric..

[25] Mlcek J., Plaskova A., Jurikova T., Sochor J., Baron M., Ercisli S. (2021). Chemical, nutritional and sensory characteristics of six ornamental edible flowers species. Foods.

[26] Rop O., Mlcek J., Jurikova T., Neugebauerova J., Vabkova J. (2012). Edible flowers—A new promising source of mineral elements in human nutrition. Molecules.

[27] Janarny G., Gunathilake K.D.P.P., Ranaweera K.K.D.S. (2021). Nutraceutical potential of dietary phytochemicals in edible flowers—A review. J. Food Biochem..

[28] Mlcek J., Rop O. (2011). Fresh edible flowers of ornamental plants—A new source of nutraceutical foods. Trends Food Sci. Technol..

[29] Mrázková M., Sumczynski D., Orsavová J. (2021). Non-traditional muesli mixtures supplemented by edible flowers: Analysis of nutritional composition, phenolic acids, flavonoids and anthocyanins. Plant Foods Hum. Nutr..

[30] Lim T.K. (2012). The Edible Medicinal and Non-Medicinal Plants. In *Edible Medicinal and Non-Medicinal Plants*. Edible Medicinal and Non-Medicinal Plants.

[31] Mitchell K.A., Markham K.R., Boase M.R. (1998). Pigment chemistry and colour of *Pelargonium* flowers. Phytochemistry.

[32] Shunying Z., Yang Y., Huaidong Y., Yue Y., Guolin Z. (2005). Chemical composition and antimicrobial activity of the essential oils of *Chrysanthemum indicum*. J. Ethnopharmacol..

[33] López-Agama I., Ramos-García M.D.L., Zamilpa A., Bautista-Baños S., Ventura-Aguilar R.I. (2021). Comparative analysis of the antioxidant compounds of raw edible flowers and ethanolic extracts of *Cucurbita pepo*, *Tagetes erecta*, and *Erythrina americana* during storage. J. Food Process. Preserv..

[34] Kaisoon O., Konczak I., Siriamornpun S. (2012). Potential health enhancing properties of edible flowers from Thailand. Food Res. Int..

[35] Jalili C., Taghadosi M., Pazhouhi M., Bahrehmand F., Miraghaee S.S., Pourmand D., Rashidi I. (2020). An overview of therapeutic potentials of *Taraxacum officinale* (dandelion): A traditionally valuable herb with a rich historical background. World Cancer Res. J..

[36] González-Castejón F., Visioli A., Rodriguez-Casado A. (2012). Diverse biological activities of dandelion. Nutr. Rev..

[37] Demasi S., Caser M., Donno D., Ravetto Enri S., Lonati M., Scariot V. (2021). Exploring wild edible flowers as a source of bioactive compounds: New perspectives in horticulture. Folia Hortic..

[38] Wu Q., Fu X., Chen Z., Wang H., Wang J., Zhu Z., Zhu G. (2022). Composition, color stability and antioxidant properties of betalain-based extracts from bracts of *Bougainvillea*. Molecules.

[39] Usami A., Kashima Y., Marumoto S., Miyazawa M. (2013). Characterization of aroma-active compounds in dry flower of *Malva sylvestris* L. by GC-MS-O analysis and OAV calculations. J. Oleo Sci..

[40] Gupta A., Sharma S., Rajput D., Patil U.K. (2024). Medicinal and culinary importance of edible flowers of Indian origin: An in-depth review. Discov. Food.

[41] Yadav R., Waghmare R. (2022). Consumer perception towards edible flower and safety issues. Indian Food Ind. Mag..

[42] Benvenuti S., Bortolotti E., Maggini R. (2016). Antioxidant power, anthocyanin content and organoleptic performance of edible flowers. Sci. Hortic..

[43] Cheng Y., Han L., Shao L., Wang H., Guo Z., Li G. (2024). Comparative investigation on the aroma profiles of edible citrus flowers in the main organs and different developmental stages. Food Chem. X.

[44] Mulík S., Hernández-Carrión M., Pacheco-Pantoja S.E., Ozuna C. (2024). Endemic edible flowers in the Mexican diet: Understanding people’s knowledge, consumption, and experience. Future Foods.

[45] Contreras-López E., Ramirez-Godinez J., García-Martínez M.M., Gutiérrez-Salomón A.L., Gonzalez-Olivares L.G., Jaimez-Ordaz J. (2021). Low-calorie beverages made from medicinal plants, flowers and fruits: Characteristics and liking of a population with overweight and obesity. Appl. Sci..

[46] Lakshan S.A.T., Jayanath N.Y., Abeysekera W.P.K.M., Abeysekera W.K.S.M. (2019). A commercial potential blue pea (*Clitoria ternatea* L.) flower extract incorporated beverage having functional properties. Evid.-Based Complement. Altern. Med..

[47] Azima A.S., Noriham A., Manshoor N. (2017). Phenolics, antioxidants and color properties of aqueous pigmented plant extracts: *Ardisia colorata* var. elliptica, *Clitoria ternatea*, *Garcinia mangostana* and *Syzygium cumini*. J. Funct. Foods.

[48] Abbasi A., Sabahi S., Bazzaz S., Tajani A.G., Lahouty M., Aslani R., Hosseini H. (2023). An edible coating utilizing *Malva sylvestris* seed polysaccharide mucilage and postbiotic from *Saccharomyces cerevisiae* var. *boulardii* for the preservation of lamb meat. Int. J. Biol. Macromol..

[49] Gokhale S., Dubey G., Khandave P., Kshirsagar S. (2018). Extraction of mucilage as a binder from the petals of *Hibiscus rosasinensis* Linn and its comparative evaluation–In vitro. Am. J. PharmTech Res..

[50] Motti R., Paura B., Cozzolino A., Falco B.D. (2022). Edible flowers used in some countries of the Mediterranean basin: An ethnobotanical overview. Plants.

[51] Egebjerg M.M., Olesen P.T., Eriksen F.D., Ravn-Haren G., Bredsdorff L., Pilegaard K. (2018). Are wild and cultivated flowers served in restaurants or sold by local producers in Denmark safe for the consumer?. Food Chem. Toxicol..

[52] Saleem H., Zengin G., Ahmad I., Lee J.T.B., Htar T.T., Mahomoodally F.M., Ahemad N. (2019). Multidirectional insights into the biochemical and toxicological properties of *Bougainvillea glabra* (Choisy.) aerial parts: A functional approach for bioactive compounds. J. Pharm. Biomed. Anal..

[53] Xiong L., Yang J., Jiang Y., Lu B., Hu Y., Zhou F., Shen C. (2014). Phenolic compounds and antioxidant capacities of 10 common edible flowers from China. J. Food Sci..

[54] Sutakwa A., Nadia L., Suharman S. (2021). Addition of blue pea flower (*Clitoria ternatea* L.) extract increases antioxidant activity in yogurt from various types of milk. J. Agercolere.

[55] Lestari P.D., Kawiji K., Yulviatun A., Martien R., Muhammad D.R.A. (2021). Physical and sensory characteristics of milk and white compound chocolate added with Asian pigeonwings flower (*Clitoria ternatea*). E3S Web Conf..

[56] Hutabarat D.J.C. (2021). Chemical and physical characteristics of fermented beverages from plant-based milk with the addition of butterfly pea flower (*Clitoria ternatea* L.) extracts. IOP Conf. Ser. Earth Environ. Sci..

[57] Pasukamonset P., Pumalee T., Sanguansuk N., Chumyen C., Wongvasu P., Adisakwattana S., Ngamukote S. (2018). Physicochemical, antioxidant and sensory characteristics of sponge cakes fortified with Clitoria ternatea extract. J. Food Sci. Technol..

[58] Chusak C., Henry C.J., Chantarasinlapin P., Techasukthavorn V., Adisakwattana S. (2018). Influence of *Clitoria ternatea* flower extract on the in vitro enzymatic digestibility of starch and its application in bread. Foods.

[59] Mulík S., Hernández-Carrión M., Pacheco-Pantoja S.E., Aguilar-Ruiz N., Ozuna C. (2022). Culinary uses of Mexican edible flowers: Recipe analysis. Int. J. Gastronom. Food Sci..

[60] Guiné R.P., Florença S.G., Ferrão A.C., Bizjak M.Č., Vombergar B., Simoni N., Vieira V. (2021). Factors affecting eating habits and knowledge of edible flowers in different countries. Open Agric..

[61] Ratnam N., Naijibullah M., Ibrahim M.D. (2017). A review on Cucurbita pepo. Int. J. Pharm. Phytochem. Res..

[62] Aquino-Bolaños E.N., Urrutia-Hernández T.A., López Del Castillo-Lozano M., Chavéz-Servia J.L., Verdalet-Guzmán I. (2013). Physicochemical Parameters and Antioxidant Compounds in Edible Squash (*Cucurbita pepo*) Flower Stored under Controlled Atmospheres. J. Food Qual..

[63] Mulík S., Ozuna C. (2020). Mexican edible flowers: Cultural background, traditional culinary uses, and potential health benefits. Int. J. Gastronom. Food Sci..

[64] Ahmed N., Hanani Y.A., Ansari S.Y., Anwar S. (2016). Jasmine (*Jasminum sambac* L., Oleaceae) oils. Essential Oils in Food Preservation, Flavor and Safety.

[65] Kanlayavattanakul M., Kitsiripaisarn S., Lourith N. (2013). Aroma profiles and preferences of *Jasminum sambac* L. flowers grown in Thailand. J. Cosmet. Sci..

[66] Loizzo M.R., Pugliese A., Bonesi M., Tenuta M.C., Menichini F., Xiao J., Tundis R. (2016). Edible flowers: A rich source of phytochemicals with antioxidant and hypoglycemic properties. J. Agric. Food Chem..

[67] Srivastava J.K., Gupta S. (2009). Health promoting benefits of chamomile in the elderly population. Complementary and Alternative Therapies and the Aging Population.

[68] Raturi M., Bose D., Mehta J., Saraf D. (2023). *Rhododendron arboreum* as a sustainable food-grade natural flavouring and colouring agent. Food Hum..

[69] Postolache A.N., Veleșcu I.D., Stoica F., Crivei I.C., Arsenoaia V.N., Usturoi M.G., Constantinescu Pop C.G., Lipșa F.D., Frunză G., Simeanu D. (2024). A Clean-Label Formulation of Fortified Yogurt Based on Rhododendron Flower Powder as a Functional Ingredient. J. Food Sci..

[70] Pinakin D.J., Kumar V., Suri S., Sharma R., Kaushal M. (2020). Nutraceutical potential of tree flowers: A comprehensive review on biochemical profile, health benefits, and utilization. Food Res. Int..

[71] Srivastava P. (2012). *Rhododendron arboreum*: An overview. J. Appl. Pharm. Sci..

[72] Krishna H., Attri B.L., Kumar A. (2014). Improvised Rhododendron squash: Processing effects on antioxidant composition and organoleptic attributes. J. Food Sci. Technol..

[73] Schmitzer V., Mikulic-Petkovsek M., Stampar F. (2019). Traditional rose liqueur—A pink delight rich in phenolics. Food Chem..

[74] Barros L., Carvalho A.M., Ferreira I.C. (2010). Leaves, flowers, immature fruits and leafy flowered stems of Malva sylvestris: A comparative study of the nutraceutical potential and composition. Food Chem. Toxicol..

[75] Fernandes L., Ramalhosa E., Pereira J.A., Saraiva J.A., Casal S. (2020). Borage, camellia, centaurea and pansies: Nutritional, fatty acids, free sugars, vitamin E, carotenoids and organic acids characterization. Food Res. Int..

[76] Grzeszczuk M., Wesolowska A., Jadczak D., Jakubowska B. (2011). Nutritional value of chive edible flowers. Acta Sci. Pol. Hortorum Cultus.

[77] Grzeszczuk M., Stefaniak A., Pachlowska A. (2016). Biological value of various edible flower species. Acta Sci. Pol. Hortorum Cultus.

[78] Gargi A., Singh J., Rasane P., Kaur S., Kaur J., Kumar M., Ercisli S. (2023). Effect of drying methods on the nutritional and phytochemical properties of pumpkin flower (*Cucurbita maxima*) and its characterization. J. Food Meas. Charact..

[79] Ninama V., Shah H., Kapadia C., Italiya A., Datta R., Singh S., Singh A. (2024). Assessment of phytochemicals, nutritional compositions and metabolite profiling using GCMS–from annual edible flowers. Sci. Hortic..

[80] Guimarães R., Barros L., Carvalho A.M., Ferreira I.C. (2010). Studies on chemical constituents and bioactivity of *Rosa micrantha*: An alternative antioxidants source for food, pharmaceutical, or cosmetic applications. J. Agric. Food Chem..

[81] dos Santos Silva L.Y., da Silva Ramos A., Cavalcante D.N., Kinupp V.F., da Silva Rodrigues J.V., Ventura B.M.L., de Araújo Bezerra J. (2023). *Hibiscus acetosella*: An unconventional alternative edible flower rich in bioactive compounds. Molecules.

[82] Pires T.C., Dias M.I., Barros L., Ferreira I.C. (2017). Nutritional and chemical characterization of edible petals and corresponding infusions: Valorization as new food ingredients. Food Chem..

[83] Jakubczyk K., Koprowska K., Gottschling A., Janda-Milczarek K. (2022). Edible flowers as a source of dietary fibre (total, insoluble and soluble) as a potential athlete’s dietary supplement. Nutrients.

[84] Fomina T.I., Kukushkina T.A. (2022). Edible flowers of onions (*Allium* L.) as a source of biologically active substances. Russ. J. Bioorg. Chem..

[85] Halder S., Khaled K.L. (2022). Quantitative estimation of mineral content from edible flowers of *Allium cepa*, *Cucurbita maxima* and *Carica papaya*: A comparative study. Int. J. Pharm. Sci. Res..

[86] Araújo S., Matos C., Correia E., Antunes M.C. (2019). Evaluation of phytochemicals content, antioxidant activity and mineral composition of selected edible flowers. Qual. Assur. Saf. Crops Foods.

[87] Coyago-Cruz E., Alarcón A., Guachamin A., Méndez G., Osorio E., Heredia-Moya J., Vera E. (2024). Functional, Antioxidant, Antibacterial, and Antifungal Activity of Edible Flowers. Antioxidants.

[88] Sotelo A., López-García S., Basurto-Peña F. (2007). Content of nutrient and antinutrient in edible flowers of wild plants in Mexico. Plant Foods Hum. Nutr..

[89] Chetia I., Das A.J., Badwaik L.S. (2024). Assessment of nutritional and bioactive properties of selected edible flowers: Characterization of phenolic compounds by reversed-phase high performance liquid chromatography. J. Chromatogr. Open.

[90] Ghosh P., Rana S.S. (2021). Physicochemical, nutritional, bioactive compounds and fatty acid profiling of Pumpkin flower (*Cucurbita maxima*), as a potential functional food. SN Appl. Sci..

[91] Marchioni I., Gabriele M., Carmassi G., Ruffoni B., Pistelli L., Najar B. (2024). Phytochemical, nutritional and mineral content of four edible flowers. Foods.

[92] Grzeszczuk M., Stefaniak A., Meller E., Wysocka G. (2018). Mineral composition of some edible flowers. J. Elementol..

[93] Dobros N., Zawada K., Paradowska K. (2022). Phytochemical profile and antioxidant activity of Lavandula angustifolia and Lavandula x intermedia cultivars extracted with different methods. Antioxidants.

[94] Zheng J., Lu B., Xu B. (2021). An update on the health benefits promoted by edible flowers and involved mechanisms. Food Chem..

[95] Fredotović Ž., Puizina J. (2019). Edible Allium species: Chemical composition, biological activity and health effects. Ital. J. Food Sci..

[96] Vahdat F., Mehdizadeh T., Kazemeini H., Reale A., Kaboudari A. (2024). Physicochemical, microbial, and sensory characteristics of yogurt with Persian shallot (*Allium hirtifolium* Boiss) and probiotic bacteria. Food Sci. Nutr..

[97] Mehdizadeh T., Kaboudari A., Reale A. (2021). Stimulatory effect of *Allium ampeloprasum* L. ssp. iranicum Wendelbo on the probiotic *Bifidobacterium bifidum* in Iranian white cheese. J. Dairy Sci..

[98] Kucekova Z., Mlcek J., Humpolicek P., Rop O. (2013). Edible flowers—Antioxidant activity and impact on cell viability. Open Life Sci..

[99] Fernandes L., Casal S., Pereira J.A., Pereira E.L., Ramalhosa E., Saraiva J.A. (2017). Effect of high hydrostatic pressure on the quality of four edible flowers: *Viola× wittrockiana*, *Centaurea cyanus*, *Borago officinalis* and *Camellia japonica*. Int. J. Food Sci. Technol..

[100] Rahaman M.M., Hossain R., Herrera-Bravo J., Islam M.T., Atolani O., Adeyemi O.S., Sharifi-Rad J. (2023). Natural antioxidants from some fruits, seeds, foods, natural products, and associated health benefits: An update. Food Sci. Nutr..

[101] Halliwell B. (1996). Antioxidants in human health and disease. Annu. Rev. Nutr..

[102] Nirmal P., Kumar M., Jose A., Tomer V., Oz E., Proestos C., Zeng M., Elobeid T., Sneha K., Oz F. (2023). Major phytochemicals: Recent advances in health benefits and extraction method. Molecules.

[103] Di Cerbo A., Carnevale G., Avallone R., Zavatti M., Corsi L. (2020). Protective effects of *Borago officinalis* (Borago) on cold restraint stress-induced gastric ulcers in rats: A pilot study. Front. Vet. Sci..

[104] Li Y., Hao Y., Gao B., Geng P., Huang H., Yu L., Choe U., Liu J., Sun J., Chen P. (2019). Chemical profile and in vitro gut microbiota modulatory, anti-inflammatory and free radical scavenging properties of *Chrysanthemum morifolium* cv. Fubaiju. J. Func. Foods.

[105] Rahnavard F., Modaresi M., Farhadi H. (2015). The comparative effects of chamomile’s hydro alcoholic extract and imipramine on decreasing depression in mice. Middle East J. Fam. Med..

[106] Yazdi H., Seifi A., Changizi S., Khori V., Hossini F., Davarian A., Jand Y., Enayati A., Mazandarani M., Nanvabashi F. (2017). Hydro-alcoholic extract of *Matricaria recutita* exhibited dual anti-spasmodic effect via modulation of Ca²⁺ channels, NO and PKA2-kinase pathway in rabbit jejunum. Avicenna J. Phytomed..

[107] Choi E.K., Guo H., Choi J.K., Jang S.K., Shin K., Cha Y.S., Choi Y., Seo D.-W., Lee Y.-B., Joo S.-S. (2015). Extraction conditions of white rose petals for the inhibition of enzymes related to skin aging. Lab. Anim. Res.

[108] Shahraki M.R., Ahmadimoghadam M., Shahraki A.R. (2015). The antinociceptive effects of hydroalcoholic extract of *Borago officinalis* flower in male rats using formalin test. Basic Clin. Neurosci..

[109] Bashir S., Janbaz K.H., Jabeen Q., Gilani A.H. (2006). Studies on spasmogenic and spasmolytic activities of *Calendula officinalis* flowers. Phytother. Res..

[110] Kucekova Z., Mlcek J., Humpolicek P., Rop O., Valasek P., Saha P. (2011). Phenolic compounds from *Allium schoenoprasum*, *Tragopogon pratensis* and *Rumex acetosa* and their antiproliferative effects. Molecules.

[111] Karimi E., Oskoueian E., Karimi A., Noura R., Ebrahimi M. (2018). *Borago officinalis* L. flower: A comprehensive study on bioactive compounds and its health-promoting properties. J. Food Meas. Charact..

[112] Aliakbarlu J., Tajik H. (2012). Antioxidant and antibacterial activities of various extracts of *Borago officinalis* flowers. J. Food Proc. Preserv..

[113] Manthena S.S., Polimati H., Annam S.S.P., Hieu H.V. (2022). Hepatoprotective Properties of Starflowers from an Annual Herb, *Borago officinalis* L. (*Boraginaceae*). Int. Pharm. Acta.

[114] Mirsadraee M., Moghaddam S.K., Saeedi P., Ghaffari S. (2016). Effect of *Borago officinalis* extract on moderate persistent asthma: A phase two randomized, double blind, placebo-controlled clinical trial. Tanaffos.

[115] Avila C., Breakspear I., Hawrelak J., Salmond S., Evans S. (2020). A systematic review and quality assessment of case reports of adverse events for borage (*Borago officinalis*), coltsfoot (*Tussilago farfara*) and comfrey (*Symphytum officinale*). Fitoterapia.

[116] Krishna R.G., Sundararajan R. (2018). Cardioprotective and antioxidant effects of *Bougainvillea glabra* against isoproterenol induced myocardial necrosis in albino rats. Int. J. Phytomed..

[117] Shalini M., Aminah A., Khalid H.M., Vimala S., Katherine S., Khoo M.G.H. (2018). In-vitro antioxidant activities, phytoconstituent and toxicity evaluation of local *Bougainvillea glabra* bract (*bunga kertas*). Int. J. ChemTech Res..

[118] Pratibha J.S., Manita T.W. (2015). Antibacterial and synergistic activity of *Calendula officinalis* methanolic petal extract on *Klebsiella pneumoniae* Co-producing ESBL and AmpC Beta Lactamase. Phytomedicine.

[119] Abdel-Aziem S.H., Hassan A.M., El-Denshary E.S., Hamzawy M.A., Mannaa F.A., Abdel-Wahhab M.A. (2014). Ameliorative effects of thyme and calendula extracts alone or in combination against aflatoxins-induced oxidative stress and genotoxicity in rat liver. Cytotechnology.

[120] Shivasharan B.D., Nagakannan P., Thippeswamy B.S., Veerapur V.P. (2013). Protective effect of *Calendula officinalis* L. flowers against monosodium glutamate induced oxidative stress and excitotoxic brain damage in rats. Indian J. Clin. Biochem..

[121] Żbik K., Onopiuk A., Szpicer A., Kurek M. (2023). Comparison of the effects of extraction method and solvents on biological activities of phytochemicals from selected violet and blue pigmented flowers. J. Food Meas. Charact..

[122] Haziri A., Faiku F., Rudhani I., Mehmeti I., Motori D. (2017). Antibacterial activity of different extracts of *Centaurea cyanus* (L.) growing wild in Kosovo. Orient. J. Chem..

[123] Escher G.B., Santos J.S., Rosso N.D., Marques M.B., Azevedo L., do Carmo M.A.V., Daguer H., Molognoni L., do Prado-Silva L., Sant’Ana A.S. (2018). Chemical study, antioxidant, anti-hypertensive, and cytotoxic/cytoprotective activities of *Centaurea cyanus* L. petals aqueous extract. Food Chem. Toxicol..

[124] Sharonova N., Nikitin E., Terenzhev D., Lyubina A., Amerhanova S., Bushmeleva K., Sinyashin K. (2021). Comparative assessment of the phytochemical composition and biological activity of extracts of flowering plants of *Centaurea cyanus* L., *Centaurea jacea* L. and *Centaurea scabiosa* L. Plants.

[125] Kim C., Kim M.C., Kim S.M., Nam D., Choi S.H., Kim S.H., Ahn K.S., Lee E.H., Jung S.H., Ahn K.S. (2013). *Chrysanthemum indicum* L. extract induces apoptosis through suppression of constitutive STAT3 activation in human prostate cancer DU145 cells. Phytother. Res..

[126] Lee M.S., Kim Y. (2020). *Chrysanthemum morifolium* flower extract inhibits adipogenesis of 3T3-L1 cells via AMPK/SIRT1 pathway activation. Nutrients.

[127] Sugawara T., Igarashi K. (2009). Identification of major flavonoids in petals of edible chrysanthemum flowers and their suppressive effect on carbon tetrachloride-induced liver injury in mice. Food Sci. Technol. Res..

[128] Jeong S.C., Kim S.M., Jeong Y.T., Song C.H. (2013). Hepatoprotective effect of water extract from *Chrysanthemum indicum* L. flower. Chin. Med..

[129] Yang P.F., Yang Y.N., Feng Z.M., Jiang J.S., Zhang P.C. (2019). Six new compounds from the flowers of *Chrysanthemum morifolium* and their biological activities. Bioorg. Chem..

[130] Oehme F.W. (1978). The hazard of plant toxicities to the human population. Effects of Poisonous Plants on Livestock.

[131] Li L., Gu L., Chen Z., Wang R., Ye J., Jiang H. (2010). Toxicity study of ethanolic extract of *Chrysanthemum morifolium* in rats. J. Food Sci..

[132] Pieroni A., Giusti M.E., Quave C.L. (2011). Cross-cultural ethnobiology in the Western Balkans: Medical ethnobotany and ethnozoology among Albanians and Serbs in the Pešter Plateau, Sandžak, South-Western Serbia. Hum. Ecol..

[133] Phrueksanan W., Yibchok-anun S., Adisakwattana S. (2014). Protection of *Clitoria ternatea* flower petal extract against free radical-induced hemolysis and oxidative damage in canine erythrocytes. Res. Vet. Sci..

[134] Verma P.R., Itankar P.R., Arora S.K. (2013). Evaluation of antidiabetic, antihyperlipidemic and pancreatic regeneration potential of aerial parts of *Clitoria ternatea*. Rev. Bras. Farmacogn..

[135] Taranalli A.D., Cheeramkuzhy T.C. (2000). Influence of *Clitoria ternatea* extracts on memory and central cholinergic activity in rats. Pharm. Biol..

[136] Morittu V.M., Musco N., Mastellone V., Bonesi M., Britti D., Infascelli F., Loizzo M.R., Tundis R., Sicari V., Tudisco R. (2021). In vitro and in vivo studies of *Cucurbita pepo* L. flowers: Chemical profile and bioactivity. Nat. Prod. Res..

[137] Maciel L.G., do Carmo M.A.V., Azevedo L., Daguer H., Molognoni L., de Almeida M.M., Granato D., Rosso N.D. (2018). *Hibiscus sabdariffa* anthocyanins-rich extract: Chemical stability, in vitro antioxidant and antiproliferative activities. Food Chem. Toxicol..

[138] Sogo T., Terahara N., Hisanaga A., Kumamoto T., Yamashiro T., Wu S., Wu S., Sakao K., Hou D.X. (2015). Anti-inflammatory activity and molecular mechanism of delphinidin 3-sambubioside, a *Hibiscus* anthocyanin. BioFactors.

[139] Nwachukwu D.C., Aneke E., Nwachukwu N.Z., Obika L.F.O., Nwagha U.I., Eze A.A. (2015). Effect of *Hibiscus sabdariffa* on blood pressure and electrolyte profile of mild to moderate hypertensive Nigerians: A comparative study with hydrochlorothiazide. Niger. J. Clin. Pract..

[140] Nwachukwu D.C., Aneke E., Obika L.F.O., Nwachukwu N.Z. (2015). Investigation of antihypertensive effectiveness and tolerability of *Hibiscus sabdariffa* in mild to moderate hypertensive subjects in Enugu, South-east, Nigeria. Am. J. Phytomed. Clin. Ther..

[141] Kim M.S., Kim J.K., Kim H.J., Moon S.R., Shin B.C., Park K.W., Yang H.O., Kim S.M., Park R. (2003). Hibiscus extract inhibits the lipid droplet accumulation and adipogenic transcription factors expression of 3T3-L1 preadipocytes. J. Altern. Complement. Med..

[142] Fakeye T.O., Pal A., Bawankule D.U., Yadav N.P., Khanuja S.P.S. (2009). Toxic effects of oral administration of extracts of dried calyx of *Hibiscus sabdariffa* Linn. (Malvaceae). Phytother. Res..

[143] Onyenekwe P.C., Ajani E.O., Ameh D.A., Gamaniel K.S. (1999). Antihypertensive effect of roselle (*Hibiscus sabdariffa*) calyx infusion in spontaneously hypertensive rats and a comparison of its toxicity with that in Wistar rats. Cell Biochem. Funct..

[144] Akindahunsi A.A., Olaleye M.T. (2003). Toxicological investigation of aqueous-methanolic extract of the calyces of *Hibiscus sabdariffa* L. J. Ethnopharmacol..

[145] Kolawole J.A., Maduenyi A. (2004). Effect of zobo drink (*Hibiscus sabdariffa* water extract) on the pharmacokinetics of acetaminophen in human volunteers. Eur. J. Drug Metab. Pharmacokinet..

[146] Kalaiselvi M., Narmadha R., Ragavendran P., Ravikumar G., Gomathi D., Sophia D., Arul Raj C., Uma C., Kalaivani K. (2011). In vivo and in vitro antitumor activity of *Jasminum sambac* (Linn) Ait Oleaceae flower against Dalton’s ascites lymphoma induced Swiss albino mice. Int. J. Pharm. Pharm. Sci..

[147] Rakhmawati A. (2022). Antimicrobial Activity and Chemical Composition Analysis of *Jasminum sambac* L. and *Plumeria alba* L. Flower Extracts. Trop. J. Nat. Prod. Res..

[148] Suaputra V., Limanan D., Yulianti E., Ferdinal F. (2021). Phytochemical Screening, Total Antioxidant Capacity and Toxicity Test of White Jasmine Flower Extract (Jasminum sambac). Proceedings of the 1st Tarumanagara International Conference on Medicine and Health (TICMIH 2021).

[149] Kunhachan P., Banchonglikitkul C., Kajsongkram T., Khayungarnnawee A., Leelamanit W. (2012). Chemical composition, toxicity and vasodilatation effect of the flowers extract of *Jasminum sambac* (L.) Ait. “G. Duke of Tuscany”. Evid.-Based Complement. Altern. Med..

[150] Nowicka P., Wojdyło A. (2019). Anti-hyperglycemic and anticholinergic effects of natural antioxidant contents in edible flowers. Antioxidants.

[151] Araj-Khodaei M., Noorbala A.A., Yarani R., Emadi F., Emaratkar E., Faghihzadeh S., Parsian Z., Alijaniha F., Kamalinejad M., Naseri M. (2020). A double-blind, randomized pilot study for comparison of Melissa officinalis L. and Lavandula angustifolia Mill. with Fluoxetine for the treatment of depression. BMC Complement. Med. Ther..

[152] FDA Code of Federal Regulations, Title 21, Part 182.10—Substances Generally Recognized as Safe. U.S. Food and Drug Administration. https://www.ecfr.gov/current/title-21/chapter-I/subchapter-B/part-182.

[153] Razavi S.M., Zarrini G., Molavi G., Ghasemi G. (2011). Bioactivity of *Malva sylvestris* L., a medicinal plant from Iran. Iran. J. Basic Med. Sci..

[154] Cheng C.L., Wang Z.Y. (2006). Bacteriostatic activity of anthocyanin of Malva sylvestris. J. For. Res..

[155] Bonjar S. (2004). Evaluation of antibacterial properties of some medicinal plants used in Iran. J. Ethnopharmacol..

[156] Beghdad M.C., Benammar C., Bensalah F., Sabri F.Z., Belarbi M., Chemat F. (2014). Antioxidant activity, phenolic and flavonoid content in leaves, flowers, stems and seeds of mallow (*Malva sylvestris* L.) from North Western of Algeria. Afr. J. Biotechnol..

[157] Seiberg M., Stone V., Iotsova V., Zhao R., Bruning E. (2010). Ingestible Compositions Containing Extracts. U.S. Patent.

[158] Cemek M., Kağa S., Şimşek N., Büyükokuroğlu M.E., Konuk M. (2008). Antihyperglycemic and antioxidative potential of *Matricaria chamomilla* L. in streptozotocin-induced diabetic rats. J. Nat. Med..

[159] Cvetanović A., Švarc-Gajić J., Zeković Z., Jerković J., Zengin G., Gašić U., Tešić Z., Mašković P., Soares C., Barroso M.F. (2019). The influence of the extraction temperature on polyphenolic profiles and bioactivity of chamomile (Matricaria chamomilla L.) subcritical water extracts. Food Chem..

[160] Menghini L., Ferrante C., Leporini L., Recinella L., Chiavaroli A., Leone S., Pintore G., Vacca M., Orlando G., Brunetti L. (2016). An hydroalcoholic chamomile extract modulates inflammatory and immune response in HT29 cells and isolated rat colon. Phytother. Res..

[161] Ionita R., Postu P.A., Mihasan M., Gorgan D.L., Hancianu M., Cioanca O., Hritcu L. (2018). Ameliorative effects of *Matricaria chamomilla* L. hydroalcoholic extract on scopolamine-induced memory impairment in rats: A behavioral and molecular study. Phytomedicine.

[162] Hemmati A.A., Jalali A., Keshavarz P. (2018). Effect of chamomile hydroalcoholic extract on Bleomycin-induced pulmonary fibrosis in rat. Tanaffos.

[163] Emami Aref P., Khoshdel A., Nicknia S., Mahmoodi M., Hajizadeh M.R., Mirzaiey M.R., Fahmidehkar M.A. (2024). Effect of Hydroalcoholic Extract of Chamomile, Aloe Vera, and Green Tea on the Diabetic Wound in Rats. Proc. Natl. Acad. Sci. India Sect. B Biol. Sci..

[164] Velusami C.C., Agarwal A., Mookambeswaran V. (2013). Effect of *Nelumbo nucifera* petal extracts on lipase, adipogenesis, adipolysis, and central receptors of obesity. Evid.-Based Complement. Altern. Med..

[165] Islam D., Huque A., Mohanta L.C., Das S.K., Sultana A., Lipy E.P., Prodhan U.K. (2018). Hypoglycemic and hypolipidemic effects of *Nelumbo nucifera* flower in Long-Evans rats. J. Herbmed Pharmacol..

[166] Wongwattanasathien O., Kangsadalampai K., Tongyonk L. (2010). Antimutagenicity of some flowers grown in Thailand. Food Chem. Toxicol..

[167] Parcha V., Yadav N., Sati A., Dobhal Y., Sethi N. (2017). Cardioprotective effect of various extract of *Rhododendron arborium* Sm flower on Albino rats. J. Pharm. Phytochem..

[168] Shi Y., Zhou M., Zhang Y., Fu Y., Li J., Yang X. (2021). Poisonous delicacy: Market-oriented surveys of the consumption of *Rhododendron* flowers in Yunnan, China. J. Ethnopharmacol..

[169] Popescu R., Kopp B. (2013). The genus *Rhododendron*: An ethnopharmacological and toxicological review. J. Ethnopharmacol..

[170] Nowak R., Olech M., Pecio Ł., Oleszek W., Los R., Malm A., Rzymowska J. (2014). Cytotoxic, antioxidant, antimicrobial properties and chemical composition of rose petals. J. Sci. Food Agric..

[171] Gholamhoseinian A., Fallah H. (2009). Inhibitory effect of methanol extract of *Rosa damascena* Mill. flowers on α-glucosidase activity and postprandial hyperglycemia in normal and diabetic rats. Phytomedicine.

[172] Lee M.H., Nam T.G., Lee I., Shin E.J., Han A.R., Lee P., Lim T.G. (2018). Skin anti-inflammatory activity of rose petal extract (*Rosa gallica*) through reduction of MAPK signaling pathway. Food Sci. Nutr..

[173] Yang H., Shin Y. (2017). Antioxidant compounds and activities of edible roses (*Rosa hybrida* spp.) from different cultivars grown in Korea. Appl. Biol. Chem..

[174] Dehghan K.A., Rasooli I., Rezaee M.B., Owlia P. (2011). Antioxidative properties and toxicity of white rose extract. J. Ethnopharmacol..

[175] Jivad N., Shahraki Z.F., Naseri A.M. (2023). An Investigation of the Protective Effects of the Hydroalcoholic Extract of Persian Yellow Rose (*Rosa foetida* Herrm.) on Rats with Parkinson’s Disease Induced by 6-Hydroxydopamine. Herb. Med. J..

[176] Mileva M., Ilieva Y., Jovtchev G., Gateva S., Zaharieva M.M., Georgieva A., Najdenski H. (2021). Rose flowers—A delicate perfume or a natural healer?. Biomolecules.

[177] Vaz C.R., Benvenutti L., Goldoni F.C., Nunes R., Schneiker G.S., Rosa G.A., Santin J.R. (2024). *Tagetes erecta* L.: A traditional medicine effective in inflammatory process treatment. J. Ethnopharmacol..

[178] Santos P.C.D., Santos V.H.M.D., Mecina G.F., Andrade A.R.D., Fegueiredo P.A., Moraes V.M.O., Silva R.M.G.D. (2015). Phytotoxicity of *Tagetes erecta* L. and *Tagetes patula* L. on plant germination and growth. S. Afr. J. Bot..

[179] Chaniad P., Techarang T., Phuwajaroanpong A., Na-Ek P., Viriyavejakul P., Punsawad C. (2021). In vivo antimalarial activity and toxicity study of extracts of *Tagetes erecta* L. and *Synedrella nodiflora* (L.) Gaertn. from the Asteraceae family. Evid.-Based Complement. Alternat. Med..

[180] Jeon H.J., Kang H.J., Jung H.J., Kang Y.S., Lim C.J., Kim Y.M., Park E.H. (2008). Anti-inflammatory activity of *Taraxacum officinale*. J. Ethnopharmacol..

[181] Dong H., Qiao J., Hou S., Ran H., Sun W., Lin B., Li Y. (2024). Potentialities of *Dandelion* (*Taraxacum Mongolicum* Hand.-Mazz.) flower extracts on gastric protection against *Helicobacter pylori* and characterization of its bioactive constituents. Chem. Biodiv..

[182] Hu C., Kitts D.D. (2005). *Dandelion* (*Taraxacum officinale*) flower extract suppresses both reactive oxygen species and nitric oxide and prevents lipid oxidation in vitro. Phytomedicine.

[183] Cho S.Y., Park J.Y., Park E.M., Choi M.S., Lee M.K., Jeon S.M., Park Y.B. (2002). Alternation of hepatic antioxidant enzyme activities and lipid profile in streptozotocin-induced diabetic rats by supplementation of dandelion water extract. Clin. Chim. Acta.

[184] Hu C., Kitts D.D. (2003). Antioxidant, prooxidant, and cytotoxic activities of solvent-fractionated *dandelion* (*Taraxacum officinale*) flower extracts in vitro. J Agric. Food Chem..

[185] Cohen S.H., Yunginger J.W., Rosenberg N., Fink J.N. (1979). Acute allergic reaction after composite pollen ingestion. J. Allergy Clin. Immunol..

[186] Martinez M., Poirrier P., Chamy R., Prüfer D., Schulze-Gronover C., Jorquera L., Ruiz G. (2015). *Taraxacum officinale* and related species—An ethnopharmacological review and its potential as a commercial medicinal plant. J. Ethnopharmacol..

[187] Musolino V., Marrelli M., Perri M.R., Palermo M., Gliozzi M., Mollace V., Conforti F. (2022). *Centranthus ruber* (L.) DC. and *Tropaeolum majus* L.: Phytochemical profile, in vitro anti-denaturation effects and lipase inhibitory activity of two ornamental plants traditionally used as herbal remedies. Molecules.

[188] Ailane L., Djahoudi A., Bennadja S. (2022). Comparative study evaluating phytochemical screening, functional groups analysis, and antimicrobial activity of *Tropaeolum majus* L. leaves, flowers, and fruits. Plant Arch..

[189] Kim G.C., Kim J.S., Kim G.M., Choi S.Y. (2017). Anti-adipogenic effects of *Tropaeolum majus* (nasturtium) ethanol extract on 3T3-L1 cells. Food Nutr. Res..

[190] Koriem K.M., Arbid M.S., El-Gendy N.F. (2010). The protective role of *Tropaeolum majus* on blood and liver toxicity induced by diethyl maleate in rats. Toxicol. Mech. Methods.

[191] Sagdic O., Ekici L., Ozturk I., Tekinay T., Polat B., Tastemur B., Bayram O., Senturk B. (2013). Cytotoxic and bioactive properties of different color tulip flowers and degradation kinetic of tulip flower anthocyanins. Food Chem. Toxicol..

[192] Koike A., Barreira J.C., Barros L., Santos-Buelga C., Villavicencio A.L., Ferreira I.C. (2015). Edible flowers of *Viola tricolor* L. as a new functional food: Antioxidant activity, individual phenolics and effects of gamma and electron-beam irradiation. Food Chem..

[193] González-Barrio R., Periago M.J., Luna-Recio C., Garcia-Alonso F.J., Navarro-González I. (2018). Chemical composition of the edible flowers, pansy (*Viola wittrockiana*) and snapdragon (*Antirrhinum majus*) as new sources of bioactive compounds. Food Chem..

[194] Moliner C., Barros L., Dias M.I., Reigada I., Ferreira I.C.F.R., López V., Langa E., Gómez Rincón C. (2019). *Viola cornuta* and *Viola* x *wittrockiana*: Phenolic compounds, antioxidant and neuroprotective activities on *Caenorhabditis elegans*. J. Food Drug Anal..

[195] Pires E.D.O., Di Gioia F., Rouphael Y., Ferreira I.C., Caleja C., Barros L., Petropoulos S.A. (2021). The Compositional Aspects of Edible Flowers as an Emerging Horticultural Product. Molecules.

[196] Takahashi J.A., Rezende F.A.G.G., Moura M.A.F., Dominguete L.C.B., Sande D. (2020). Edible Flowers: Bioactive Profile and Its Potential to Be Used in Food Development. Food Res. Int..

[197] Pigłowski M., Niewczas-Dobrowolska M. (2023). Hazards in products of plant origin reported in the rapid alert system for food and feed (RASFF) from 1998 to 2020. Sustainability.

[198] Wilczynska A., Kukułowicz A., Lewandowska A. (2021). Preliminary assessment of microbial quality of edible flowers. LWT—Food Sci. Technol..

[199] Rawat S. (2015). Food spoilage: Microorganisms and their prevention. Asian J. Plant Sci. Res..

[200] Kowalska B., Szczech M. (2022). Differences in microbiological quality of leafy green vegetables. Ann. Agric. Environ. Med..

[201] Habibi Najafi M.B., Bahreini M. (2012). Microbiological quality of mixed fresh-cut vegetable salads and mixed ready-to-eat fresh herbs in Mashhad, Iran. Proceedings of the International Conference on Nutrition and Food Sciences IPCBEE.

[202] Tournas V.H. (2005). Moulds and yeasts in fresh and minimally processed vegetables, and sprouts. Int. J. Food Microbiol..

[203] Lee Y.-S., Lee D.-H., Hwang E.-K., Sohn H.-Y. (2022). Monitoring of Pathogenic Bacteria, Heavy Metals, and Pesticide Residues in Commercial Edible Dry Flowers. J. Life Sci..

[204] Purohit S.R., Rana S.S., Idrishi R., Sharma V., Ghosh P. (2021). A review on nutritional, bioactive, toxicological properties and preservation of edible flowers. Future Foods.

[205] Wilczyńska A., Kukułowicz A., Lewandowska A. (2023). Effect of Packaging on Microbial Quality of Edible Flowers During Refrigerated Storage. Polish J. Food Nutr. Sci..

[206] Fernandes L., Casal S., Pereira J.A., Pereira E.L., Saraiva J.A., Ramalhosa E. (2020). Freezing of edible flowers: Effect on microbial and antioxidant quality during storage. J. Food Sci..

[207] Polizzi D., Aiello V., Guarnaccia A., Vitale G., Perrone G., Stea G. (2010). First Report of Fusarium Wilt of Paper Flower (*Bougainvillea glabra*) Caused by *Fusarium oxysporum* in Italy. Plant Dis..

[208] Fürnkranz M., Lukesch B., Müller H., Huss H., Grube M., Berg G. (2012). Microbial Diversity Inside Pumpkins: Microhabitat-Specific Communities Display a High Antagonistic Potential Against Phytopathogens. Microb. Ecol..

[209] Sasu I., Seidl-Adams K., Wall J.A., Winsor A.G., Stephenson A.G. (2010). Floral Transmission of *Erwinia tracheiphila* by Cucumber Beetles in a Wild *Cucurbita pepo*. Environ. Entomol..

[210] Baruzzi F., Cefola M., Carito A., Vanadia S., Calabrese N. (2012). Changes in Bacterial Composition of Zucchini Flowers Exposed to Refrigeration Temperatures. Sci. World J..

[211] Lara-Cortés S., Bautista-Baños L., Barrera-Necha L.L., Hernández-Zárate G., León-Rodríguez R. (2019). Detección e identificación molecular de *Pantoea vagans* en flores de *Dahlia* sp. TIP Rev. Especializada en Cienc. Químico-Biol..

[212] Park J.H., Cho S.E., Han K.S., Lee S.H., Shin H.D. (2014). First Report of *Choanephora* Blight Caused by *Choanephora infundibulifera* on *Hibiscus rosa-sinensis* in Korea. Plant Dis..

[213] Seidler-Lozykowska K., Mordalski R., Kucharski W., Kedzia B., Bocianowski J. (2014). Yielding and Quality of Lavender Flowers (*Lavandula angustifolia* Mill.) From Organic Cultivation. Acta Sci. Pol. Hortorum Cultus.

[214] Kuang W., Gong X., Lin Y., Chen L., Zheng X., Tang J., Shi X., Sun X., Zhang L., Cui R. (2023). First Report of *Serratia marcescens* Causing Seed Necrosis on *Nelumbo nucifera* in China. Crop Prot..

[215] Chaudhari D., Kiran S., Choudhary A., Silveira K., Narwade N., Dhotre D., Khazir J., Mir B.A., Shouche Y.S., Rahi P. (2022). Prokaryotic Communities Adapted to Microhabitats on the Indian Lotus (*Nelumbo nucifera*) Growing in the High-Altitude Urban Dal Lake. Int. Microbiol..

[216] Carpena M.A., Prieto M., Trząskowska M. (2024). Chemical and Microbial Risk Assessment of Wild Edible Plants and Flowers. EFSA J..

[217] Wetzel K., Lee J., Lee C.S., Binkley M. (2010). Comparison of Microbial Diversity of Edible Flowers and Basil Grown with Organic Versus Conventional Methods. Can. J. Microbiol..

[218] Ampuero J., Latorre B.A., Torres R., Chávez E.R. (2008). Identification of Phytophthora cryptogea as the Cause of Rapid Decline of Petunia (Petunia × hybrida) in Chile. Plant Dis..

[219] South K.A., Peduto Hand F., Jones M.L. (2020). Beneficial Bacteria Identified for the Control of *Botrytis cinerea* in Petunia Greenhouse Production. Plant Dis..

[220] Xue W., Macleod J., Blaxland J. (2023). The Use of Ozone Technology to Control Microorganism Growth, Enhance Food Safety and Extend Shelf Life: A Promising Food Decontamination Technology. Foods.

[221] (2010). European Pharmacopoeia.

[222] Ruiz Rodríguez L.G., Mohamed F., Bleckwedel J., Medina R., Vuyst L.D., Hebert E.M., Mozzi F. (2019). Diversity and Functional Properties of Lactic Acid Bacteria Isolated from Wild Fruits and Flowers Present in Northern Argentina. Front. Microbiol..

[223] Hussein Z., Caleb O.J., Opara U.L. (2015). Perforation-Mediated Modified Atmosphere Packaging of Fresh and Minimally Processed Produce—A Review. Food Packag. Shelf Life.

[224] Demasi S., Mellano M.G., Falla N.M., Caser M., Scariot V. (2021). Sensory Profile, Shelf Life, and Dynamics of Bioactive Compounds During Cold Storage of 17 Edible Flowers. Horticulturae.

